# *SMARCB1*-related schwannomatosis and other *SMARCB1*-associated phenotypes: clinical spectrum and molecular pathogenesis

**DOI:** 10.1007/s10689-025-00486-4

**Published:** 2025-08-12

**Authors:** Hildegard Kehrer-Sawatzki, David N. Cooper

**Affiliations:** 1 Institute of Human Genetics, University of Ulm, Albert-Einstein-Allee 11, 89081 Ulm, Germany; 2 Institute of Medical Genetics, School of Medicine, Cardiff University, Cardiff, CF14 4XN UK

## Abstract

SMARCB1 is a core unit of the BAF chromatin remodelling complex and its functional impairment interferes with the self-renewal and pluripotency of stem cells, lineage commitment, cellular identity and differentiation. *SMARCB1* is also an important tumour suppressor gene and somatic *SMARCB1* pathogenic variants (PVs) have been detected in ~ 5% of all human cancers. Additionally, germline *SMARCB1* PVs have been identified in patients with conditions as clinically diverse as Rhabdoid Tumour Predisposition Syndrome type 1 (RTPS1), schwannomatosis and neurodevelopmental disorders such as Coffin-Siris syndrome (CSS). RTPS1 is characterized by the occurrence of highly malignant atypical teratoid rhabdoid tumours (AT/RT) affecting mostly infants, whereas *SMARCB1*-related schwannomatosis is generally diagnosed after the age of 30 and is characterized by benign schwannomas. Patients with germline *SMARCB1* PVs and neurodevelopmental disorders do not usually develop *SMARCB1*-deficient tumours but instead exhibit severe intellectual disability and congenital malformations. It is intriguing how germline *SMARCB1* PVs can be responsible for these very different pathologies. However, a network of different factors has emerged that play important roles in this context. Thus, the tumour phenotype associated with germline *SMARCB1* PVs is determined by the nature and location of the *SMARCB1* mutation and the timing of *SMARCB1* inactivation in specific progenitor cells. Biallelic complete loss of SMARCB1 function during a narrow time window of early embryonic development in neural crest cells is essential for AT/RT development. By contrast, hypomorphic *SMARCB1* PVs during later developmental stages affecting more differentiated Schwann cell precursors give rise to schwannomas. However, the loss of the wild-type *SMARCB1* allele is insufficient for schwannoma growth which appears to be dependent upon concomitant somatic *NF2* PVs in patients with *SMARCB1*-related schwannomatosis according to the four-hit/three-step model of tumorigenesis. In patients with neurodevelopmental disorders such as CSS, germline PVs would appear to cluster within the C-terminal *SMARCB1* domain, interfering with the nucleosomal interactions of SMARCB1 but not with its tumour suppressor activity.

**Supplementary Information:**

The online version contains supplementary material available at 10.1007/s10689-025-00486-4.

## Introduction

The protein product of the *SMARCB1* gene (also known as hSNF5, INI1 and BAF47) is a core member of the mammalian SWItch/Sucrose Non-Fermentable (SWI/SNF) complex, also termed the BAF (Brg/Brahma-associated factor) chromatin remodelling complex. The human *SMARCB1* gene (MIM #601607) was originally cloned as the homolog of yeast SWI/SNF complex member *SNF5* [1]. Subsequently, *SMARCB1* was found to act as a *bona fide* tumour suppressor in malignant rhabdoid tumours (MRTs) and many other tumour types. Indeed, both inherited (germline) and acquired (somatic) *SMARCB1* mutations have been implicated in causing the highly aggressive intracranial atypical teratoid/rhabdoid tumour (AT/RT) [2–8, reviewed in 9]. AT/RTs are characterized by the biallelic loss of *SMARCB1* function [2, 10] and are highly malignant, developing in the main in infants and very young children, frequently leading to death within the first few years of life [reviewed by 11]. Approximately 25–35% of patients with AT/RT carry a germline *SMARCB1* alteration that defines the Rhabdoid Tumour Predisposition Syndrome type 1 (RTPS1; MIM #609322) [12, 13, reviewed by 14]. AT/RTs are intracranial MRTs but MRTs may also arise in extracranial tissues such as kidney or soft tissues. In many instances, MRT develop before the age of three [15]. Less frequently, MRTs may also arise in adults [reviewed by 16].

*SMARCB1* functions as a classical tumour suppressor in cases of MRT and complete loss of nuclear SMARCB1 protein expression is characteristic of this type of malignancy [reviewed by 17]. In addition to MRTs, many other tumour types exhibit somatic *SMARCB1* gene inactivation or loss of expression; this group of malignancies has been collectively defined as *SMARCB1*-deficient tumours [reviewed by 18–20]. Astonishingly, 5% of all human cancers have pathogenic variants (PVs), albeit mostly somatic, in the *SMARCB1* gene [21] highlighting its general importance in tumorigenesis.

The SMARCB1 protein is an important component of the BAF complexes, which are chromatin remodelers comprising multiple subunits mobilizing nucleosomes and regulating gene expression [22, reviewed by 20]. It turns out that approximately 20% of all human cancers have mutations in one of the BAF complex subunits [18]. The analysis of MRTs and other *SMARCB1*-deficient malignant tumours has indicated the consequences of complete SMARCB1 protein loss including profound changes in epigenetic architecture, aberrant activation of transcriptional and metabolic programs that promote cell growth, deregulation of stem cell maintenance and suppression of terminal differentiation [23–26]. In *SMARCB1*-deficient malignancies, the dysregulation of the BAF complex-dependent chromatin remodelling machinery leads to reprogramming and a blockage of differentiation that drives these cells to malignancy [27].

In 2007, germline pathogenic *SMARCB1* variants were identified for the first time as predisposing to familial schwannomatosis [28]. This came as some surprise since schwannomatosis is characterized by the occurrence of mainly benign tumours and a median age at diagnosis of 40 years (range, 16–70 years) [29]. This is in stark contrast to the involvement of *SMARCB1* in the tumorigenesis of highly malignant aggressive tumours such as pediatric AT/RT associated with a very poor prognosis. Non-*NF2*-related schwannomatosis is characterized by the development of multiple benign schwannomas of the spinal, peripheral and cranial nerves in the absence of intra-dermal schwannomas, ependymomas and ophthalmic features [29–32]. Since the first discovery of *SMARCB1* PVs causing late-onset Schwann cell-derived tumours, it became clear that *SMARCB1*-related schwannomatosis (SWN) is one of the major forms of schwannomatosis [33–43]. The fact that germline *SMARCB1* PVs not only predispose to SWN but also to highly malignant pediatric tumours in the context of RTPS1, indicates that cells of different origin must be vulnerable to the complex cellular, molecular and developmental disturbances resulting from *SMARCB1* loss. In addition to its role as a tumour suppressor, *SMARCB1* also plays an important role during neurodevelopment [reviewed by 44]. Thus, germline PVs in *SMARCB1* may also cause neurodevelopmental disorders associated with severe intellectual disability such as Coffin-Siris syndrome (CSS, MIM #135900), which is not associated with the development of pediatric malignancies such as MRTs [45–49]. In turn, severe intellectual disability is not observed in patients with *SMARCB1*-related SWN or in carriers of germline *SMARCB1* PVs in RTPS1 families. To make matters even more complicated, in families with RTPS1, carriers of pathogenic *SMARCB1* variants have been identified without clinical symptoms. In most of these families, mosaicism cannot account for the lack of penetrance [8, 33, 50–56].

The different pathologies associated with germline *SMARCB1* PVs are likely to be caused by a number of different determinants including the type of pathogenic *SMARCB1* variant and its position within the different regions/domains of the gene/protein, the timing of the loss of the second *SMARCB1* allele, the type of mutation associated with the loss of the second *SMARCB1* allele (intragenic PV, large deletion, loss of chromosome 22q), the cellular origin of the tumour progenitor cells and the possible concomitant loss of other tumour suppressor genes. Furthermore, complex epigenetic and transcriptome changes caused by *SMARCB1* mutation may play an important role in defining the clinical phenotype associated with *SMARCB1* loss.

**Fig. 1:**
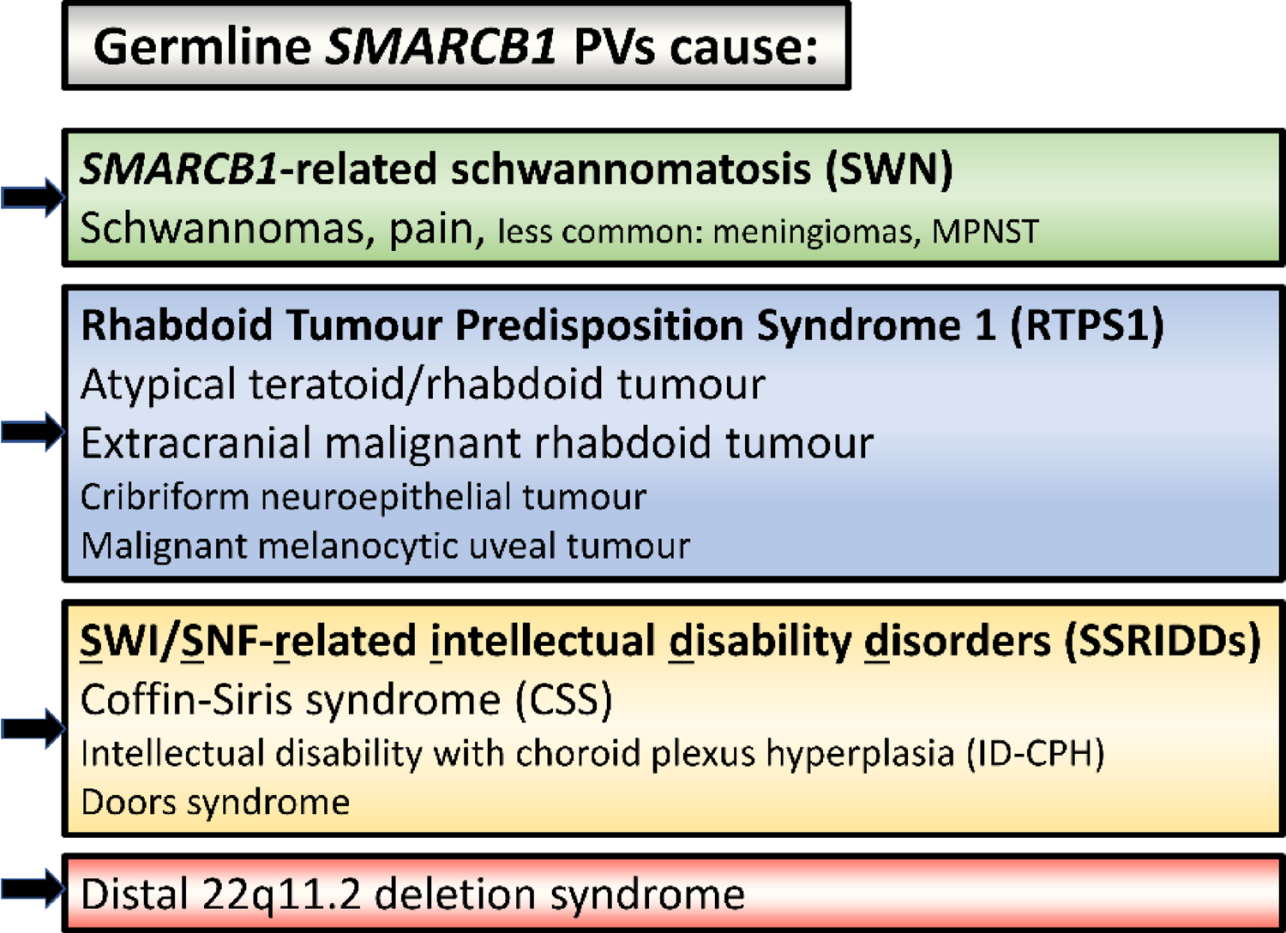
Pathologies associated with germline *SMARCB1* pathogenic variants (PVs)

This review focuses on the germline pathogenic *SMARCB1* variants responsible for a number of completely different diseases including schwannomatosis, RTPS1 and syndromic neurodevelopmental disorders (Fig. 1) as well as the functional impact of *SMARCB1* loss in the context of these very different pathologies. Furthermore, particular attention is paid to the pathogenic consequences of *SMARCB1* loss including disturbances in cellular differentiation and lineage specification of neural crest cells underlying the tumorigenesis of either poorly differentiated pediatric rhabdoid tumours or more differentiated adult tumours such as schwannomas.

## Clinical spectrum of *SMARCB1*-related schwannomatosis and other *SMARCB1*-associated phenotypes

### Schwannomatosis (SWN)

The autosomal dominant inherited tumour predisposition syndromes, schwannomatosis (collectively termed non-*NF2*-related SWN) and neurofibromatosis type-2 (NF2) (now designated as *NF2*-related schwannomatosis), predispose affected individuals to the development of schwannomas. These benign, well-circumscribed nerve sheath tumours only very rarely undergo malignant transformation [54, 57–60]. They contain clonal populations of Schwann cells and most schwannomas are sporadic [reviewed by 61]. However, some cases are associated with a genetic predisposition and occur in the context of a form of schwannomatosis (SWN). Despite the clinical overlap between non-*NF2*-related SWN and *NF2*-related SWN, it became clear quite early on that they are distinct clinical and genetic entities since patients with non-*NF2*-related SWN do not exhibit bilateral vestibular schwannomas, which is a hallmark feature of *NF2-*related SWN [30, 32, 62]. Furthermore, patients with non-*NF2*-related SWN do not have germline pathogenic variants (PVs) in the *NF2* gene. Instead, tumour-specific PVs in the *NF2* gene characterize the schwannomas of patients with non-*NF2*-related SWN [35, 62, 63]. Until the identification of the schwannomatosis-causing genes *LZTR1* and *SMARCB1*, schwannomatosis was mainly identified by clinical criteria and by the exclusion of germline *NF2* PVs [31, 64] (Supplementary Table 1). Germline pathogenic *SMARCB1* variants were identified in 2007 as a cause of non-*NF2*-related SWN [28]. Seven years later, in 2014, *LZTR1* became the second gene to be identified as causing non-*NF2*-related SWN [65]. In view of the clinical overlap between the different types of schwannomatosis, an update of the diagnostic criteria and the nomenclature for these schwannoma predisposition syndromes became necessary, including the need for genetic testing to arrive at the correct differential diagnosis [66].

#### *SMARCB1*-related SWN in relation to other types of SWN

The term schwannomatosis comprises a group of neurogenetic disorders that differ in terms of their genetic predisposition (Table 1). The most common form of schwannomatosis is *NF2*-related SWN, which is caused by heterozygous pathogenic *NF2* gene variants. The non-*NF2* related forms of SWN are less common and include *LZTR1*-related SWN and *SMARCB1*-related SWN. Affected individuals harbour germline or first hit PVs in the *LZTR1* gene or *SMARCB1* gene, respectively (Table 1). In addition to *LZTR1* and *SMARCB1*, other SWN predisposing genes may exist since in 14–30% of patients with familial SWN, and in 60% of sporadic SWN cases, no germline PVs have been detected in *LZTR1*,* SMARCB1* or *NF2* by the application of conventional mutation screening assays [33–36, 38, 40, 67–69]. So far, only a few other genes have been identified as putative SWN-causing genes since pathogenic variants were identified in these genes in SWN patients without germline PVs in *NF2*, *SMARCB1* or *LZTR1*. However, none of them would appear to account for a significant number of SWN cases since pathogenic variants have only been identified in single patients or families [43, 70–75] (Supplementary Table 2).

**Table 1:** Classification of the different types of schwannomatosis (SWN) following Plotkin et al. [66]

SWN type (MIM #)	Gene (MIM #) [Accession number]	Specification
*NF2*-related SWN (101000)	*NF2* (101000) [NM_000268.4]	Autosomal dominant inherited syndrome caused by heterozygous germline PVs in the *NF2* gene, which is located on chromosome 22q12.2, and which encodes the Merlin protein. Mosaic *NF2*-related SWN is caused by somatic PVs in *NF2* that are not present in all cells of the patient in question.
*LZTR1*-related SWN (615670)	*LZTR1* (600574) [NM_006767.4]	Autosomal dominant condition caused by heterozygous germline PVs in the *LZTR1* gene, located on chromosome 22q11.21.
*SMARCB1*-related SWN (162091)	*SMARCB1* (601607) [NM_003073.5]	Autosomal dominant condition caused by heterozygous germline PVs in the *SMARCB1* gene, located on chromosome 22q11.23.
22q-related SWN	Unknown	Classification intended for patients with multiple schwannomas with common molecular findings on chromosome 22q such as tumour-specific loss of heterozygosity/ large (multi-gene) deletions.
SWN not otherwise specified (**NOS**)	Unknown	Classification intended for patients who exhibit clinical features of *NF2*-related SWN or non-*NF2*-related SWN but have not been subjected to molecular analysis.
SWN not elsewhere classified (**NEC**)	Unknown	Classification intended for patients in whom molecular analysis of blood and tumours has failed to detect a PV in one of the known SWN genes.

PV: pathogenic variant; SWN: schwannomatosis

Somatic mosaicism for a *SOX10* (MIM #602229) indel PV was identified in two patients with segmental SWN but they lacked germline PVs in *SMARCB1* or *LZTR1* [76]. Further, heterozygous somatic *SOX10* indel PVs were detected in 29% of sporadic non-vestibular schwannomas [77] and in 93% of sporadic gastrointestinal schwannomas [78]. These findings indicate that a subgroup of sporadic schwannomas can arise in the context of disturbed cellular differentiation of Schwann cells resulting from mutated *SOX10* [77]. So far, there are no hints as to a putative role for pathogenic *SOX10* variants during tumorigenesis in patients with *SMARCB1*-related SWN or any other type of SWN.

#### Prevalence of *SMARCB1*-related SWN and other SWN types

Epidemiological studies from the UK have indicated that *NF2*-related SWN has a prevalence of 1 in 61,332 individuals [79]. The proportion of *de novo* cases among those with *NF2*-related SWN was found to be very high (72%) [79]. The second most common type of SWN was found to be *LZTR1*-related SWN. According to the UK data, the prevalence of *LZTR1*-related SWN is 1 in 527,000 [79]. Much less common is *SMARCB1*-related SWN with a prevalence of just 1 in 1.1 million individuals [79]. Consequently, *LZTR1*- and *SMARCB1*-related SWN are 8.4–18.4 times less frequent than *NF2*-related SWN.

#### Diagnostic criteria for *SMARCB1*-related SWN

The clinical overlap between the different forms of SWN represents a challenge in differentiating these disorders [32]. This made an update of the diagnostic criteria and nomenclature for the different types of SWN both necessary and urgent [66]. Crucial to differential diagnosis in SWN is genetic testing including blood as well as tumour tissue [66, 80, 81]. Genetic testing also ensures the timely diagnosis of SWN [82]. The updated diagnostic criteria for *SMARCB1*-related SWN are listed in Table 2.

**Table 2:** Diagnostic criteria for *SMARCB1*-related SWN after Plotkin et al. [66]

A diagnosis of *SMARCB1*-related SWN may be made when an individual meets one of the following criteria:				
At least one pathologically confirmed schwannoma or hybrid nerve sheath tumour and a *SMARCB1* pathogenic variant in an unaffected tissue such as blood^a^				
A shared *SMARCB1* PV in at least two schwannomas or hybrid nerve sheath tumours				
Pattern of genetic changes in unaffected and tumour tissues in *SMARCB1*-related SWN				
Gene	Unaffected tissue^b^	Tumour 1	Tumour 2	Comment
*SMARCB1*				
Allele 1	PV1^c^	PV1	PV1	Shared *SMARCB1* pathogenic variant
Allele 2	WT	LOH	LOH	LOH typically presents as a deletion of the 22q region encompassing the *LZTR1/SMARCB1/NF2* genes
*NF2*				
Allele 1	WT	PV2	PV3	Tumour-specific pathogenic *NF2* variant in *cis* to the pathogenic *SMARCB1* variant
Allele 2	WT	LOH	LOH	Tumour-specific partial loss of 22q in the *trans* position, LOH typically presents as a deletion of the 22q region encompassing the *LZTR1/SMARCB1/NF2* genes

LOH, loss of heterozygosity; PV, pathogenic variant; WT, wild-type

^a^If a likely pathogenic variant is identified, tumour analysis may help to classify the PV

^b^Tissues unaffected by tumours e.g. blood or skin

^c^If the variant allele fraction is clearly < 50%, then the diagnosis is mosaic *SMARCB1*-related SWN

Particularly difficult can be the differential diagnosis between mosaic *NF2*-related SWN and non-*NF2*-related SWN, specifically *LZTR1*-related SWN [83, 84]. This is because unilateral vestibular schwannomas may occur in patients with mosaic *NF2*-related SWN but also in patients with germline *LZTR1* PVs (Table 3). Furthermore, some patients with mosaic *NF2*-related SWN may not develop vestibular schwannomas at all and only present with peripheral schwannomas. Additionally, *LZTR1* variant classification can be impeded by the high number of *LZTR1* loss-of-function variants in the general population [69, 85]. This might not however be as much of a problem in the context of *SMARCB1*, since this gene is highly loss-of-function intolerant [86].

#### Mosaic *NF2*-related SWN mimicking non-*NF2*-related SWN

Mosaic *NF2*-related SWN often exhibits substantial clinical overlap with non-*NF2*-related SWN resulting in the misdiagnosis of at least 9% of non-*NF2*-related SWN cases [32]. Remarkably, 57% of patients clinically diagnosed with non-*NF2*-related SWN but without germline *LZTR1* or *SMARCB1* lesions exhibit mosaic *NF2*-related SWN as determined by identical pathogenic *NF2* variants detected in two independent schwannomas [32, 84]. It should be noted that somatic mosaicism is quite frequent in *NF2*-related SWN cases, being observed in at least 33% of *de novo* patients with bilateral vestibular schwannomas and in up to 60% of *de novo* patients with unilateral vestibular schwannomas [87–90].

Mosaic *NF2*-related SWN can be difficult to identify by genetic testing without at least two tumours being available for analysis in addition to blood [32, 43, 64, 81, 89, 91, 92]. No fewer than 43% of the patients with at least one non-vestibular schwannoma, and who did not meet the clinical criteria for *NF2*-related SWN, exhibited somatic mosaicism for an *NF2* PV and hence had mosaic *NF2*-related SWN [81]. Conspicuously, 1.8% of patients with apparently sporadic vestibular schwannomas actually had mosaic *NF2*-related SWN, whilst 3% had a germline *LZTR1* PV [93]. Taken together, these findings emphasize the importance of comprehensive genetic testing of tumour and blood samples for the differential diagnosis of patients with schwannomas.

### Clinical presentation of patients with non-*NF2*-related SWN

In many studies assessing the clinical symptoms of patients with schwannomatosis (SWN), no strict distinction was made between *LZTR1*-related or *SMARCB1*-related or any other type of non-*NF2*-related SWN. Instead, the patients were analysed as a miscellaneous group having ‘schwannomatosis’. In the following, we shall refer to this genetically heterogeneous group as patients with non-*NF2*-related SWN. Whenever possible, the clinical differences between *SMARCB1*-related SWN compared to other types of SWN will be emphasized.

#### Age at initial referral, diagnosis and life expectancy

Patients with non-*NF2*-related SWN are most commonly diagnosed in adulthood [94, 95]. The median age at initial symptoms was 30 years (range: 8–59 years) and the median age at diagnosis was 40 years (range: 16–70 years) according to the study of Merker et al. [29].

Isolated schwannomas in patients with *SMARCB1-*, *LZTR1-* or *NF2*-related SWN may already be present in early childhood or in young adults [96]. In a total of 153 patients aged younger than 24 years with an isolated schwannoma, genetic testing indicated that 15 (9.8%) patients had a germline *NF2* pathogenic variant, six patients (3.9%) had a germline *SMARCB*1 PV, and 10 patients (6.5%) had a germline *LZTR1* PV [96]. A total of 13 patients (8.5%) with an isolated schwannoma had mosaic *NF2*-related SWN, while somatic mosaicism for either *SMARCB1*- or *LZTR1*-related SWN was not observed [96]. These findings indicate that both *SMARCB1*- and *LZTR1*-related schwannomatosis can present already in childhood or in young adulthood with an isolated schwannoma and should be suspected in addition to *NF2*-related SWN [97].

Remarkably, the life expectancy is significantly higher in patients with non-*NF2*-related SWN (mean age at death, 76.9) as compared to patients with *NF2*-related SWN (mean age at death, 66.2) [32]. Early age at diagnosis, truncating *NF2* PVs and the presence of intracranial meningiomas were associated with increased mortality in *NF2*-related SWN [98–100]. Further, the presence of lower cranial nerve schwannomas is a poor prognostic factor in *NF2*-related SWN [101]. In patients with *SMARCB1*-related schwannomatosis, the increased malignancy risk must be considered which may contribute to reduced life expectancy [97, 102, 103] (Sect. Malignancy risk in patients with *SMARCB1*-related SWN).

#### Pain

The most common symptom reported by patients with non-*NF2*-related *SWN* is chronic pain, affecting 67–94% of patients [29, 31, 95, 104, 105]. Pain without a visible or palpable tumour affects 35–46% of SWN patients [29, 32]. Pain associated with a schwannoma has been reported by 11% of SWN patients [29]. However, not all schwannomas are painful. In non-*NF2* SWN, a significant association was observed between high tumour volume and high levels of pain [104, 106]. SWN-associated pain can include local, multifocal or diffuse pain, which might be regarded as systemic neuropathic pain irrespective of the location of the schwannomas [29, 107]. SWN patients often have persistent or recurrent pain despite the surgical removal of schwannomas, and exhibit generalized whole-body pain [29]. There is no medication that is broadly effective in treating SWN-associated pain [108]. Surgical removal of painful schwannomas may result in complete resolution of pain symptoms but some painful schwannomas have a significant neuropathic component and drugs such as tricyclic antidepressants and gabapentinoids may help to improve the quality of life of affected patients [103]. According to the guidelines published by the European Reference Network (ERN) for Genetic Tumour Risk Syndromes (GENTURIS), the permanent use of opioids to reduce the pain in patients with schwannomatosis is not recommended owing to their poor effect on neuropathic pain and associated dependency and hyperalgesia [103].

Importantly, pain appears to correlate with the germline PV in patients with SWN. Pain-associated quality of life is significantly worse in patients with *LZTR1*-related SWN as compared to patients with *SMARCB1*-related SWN [104]. In this study, high pain levels correlated with increased whole-body tumour volume but not with the number of tumours. Further, the tumour location appears unlikely to be the primary driver of pain. Pain and pain-related quality of life were not significantly different between patients with and without spinal schwannomas [104].

##### Reduced intra-epidermal nerve fibre density in patients with non-*NF2*-related SWN

The molecular mechanisms underlying tumour-associated or neuropathic pain in patients with schwannomatosis have not so far been fully elucidated. Patients with non*-NF2*-related SWN exhibit a markedly lower intra-epidermal nerve fibre density (IEND) in skin biopsies as compared to controls [109]. The reduced IEND may reflect a reduction in C-fibres causing small fibre neuropathy associated with neuropathic pain [109]. Hence, patients with SWN may suffer from a form of small fibre neuropathy associated with chronic neuropathic pain. The study of Farschtschi et al. [109] included 15 patients with *LZTR1*-related SWN, one patient with *SMARCB1*-related SWN and 16 schwannomatosis patients without a pathogenic variant in either gene as determined by the analysis of blood samples. Misra et al. [110] analysed a cohort of 88 patients with small fibre neuropathy (SNF) and identified two patients with likely pathogenic variants in the *LZTR1* gene. Whether this is a hint that *LZTR1* plays an important role in the etiology of SNF or whether this is random co-occurrence is unclear. In this context, it should be remembered that the frequency of loss of function *LZTR1* variants in the general population is quite high (0.36%) [69].

##### Schwannomas with and without pain in non-*NF2*-related SWN

Remarkably, some schwannomas in patients with SWN are not painful, whereas others are associated with severe pain, and both types may occur in the same patient. Mansouri et al. [111] observed that a significant proportion of painful schwannomas in patients with SWN affected the lower extremities, occurring predominantly in females and particularly in those with germline PVs in *LZTR1*. Furthermore, 16% of the very painful schwannomas were positive for the somatic *SH3PXD2A-HTRA1* gene fusion [111] (Sect. Recurrent *SH3PXD2A-HTRA1* fusion in SWN-schwannomas). Notably, painful schwannomas in SWN patients exhibit a significantly upregulated RAS/MAPK pathway. This is likely to be caused by *LZTR1* deficiency in these tumours. Normally, the LZTR1 protein facilitates the polyubiquitination-mediated degradation of RAS proteins via the proteasomal degradation systems, leading to the inhibition of RAS/MAPK signalling activity [112, 113]. Further, the ERBB2/HER2 and VEGF pathways are significantly upregulated in painful schwannomas from patients with germline *LZTR1* PVs. Moreover, painful schwannomas from patients with *LZTR1*- and *SMARCB1*-related SWN have been found to contain a significantly higher proportion of mast cells than pain-free schwannomas [111]. Mast cells are known modulators of nociceptive pain [114, 115].

Remarkably, some painful schwannomas from SWN patients would appear to secrete different factors that act on nearby nerves to augment nociception by neuronal sensitization or spontaneous neuronal firing. Thus, it may be concluded that some painful schwannomas exhibit a specific ’secretome’ [116]. This has been examined using immortalized cell lines established from primary painful and non-painful schwannomas of patients with SWN [117]. Importantly, the cell lines demonstrated the same gene expression pattern as the schwannomas they were derived from, as confirmed by microarray expression analysis [117]. Conditioned medium (CM) collected from cell lines of painful schwannomas, but not that from cell lines derived from non-painful schwannomas, contained increased amounts of multiple cytokines [116]. Furthermore, culturing mouse dorsal root ganglion neurons with CM derived from painful schwannomas led to the upregulated expression of known inflammatory pain-related genes and an increased responsiveness to noxious agonists (capsaicin and/or cinnamaldehyde) of TRPV1 and TRPA1 calcium channels [116]. In this model system, substances secreted by painful schwannomas would seem to sensitize neurons and alter neuronal gene expression [116]. However, the schwannomas analysed by Ostrow et al. [116] were not classified by the type of germline pathogenic variant causing SWN. In a follow-up study, Rubright et al. [118] included the classification by germline PV and analysed cell lines established from schwannomas of patients with either *SMARCB1*-related SWN or *LZTR1*-related SWN. These authors also included schwannomas derived from patients with NEC-related SWN, in whom molecular analysis of blood and tumours had failed to detect a PV in either *LZTR1* or *SMARCB1* (Table 1). Their analysis confirmed previous findings since CM from painful schwannoma cell lines contained elevated levels of specific inflammatory cytokines (IL-6, IL-8, VEGF), compared with CM collected from cell lines derived from non-painful schwannomas. Remarkably, the CM from painful schwannoma-derived cell lines of patients with NEC-related SWN (termed NEC-CM) contained higher levels of IL-8, CCL2 and CCL20 than CM from painful schwannoma cell lines of patients with *SMARCB1*-related SWN (*SMARCB1*-CM) and CM from painful schwannoma cell lines of patients with *LZTR1*-related SWN (*LZTR1*-CM). Painful *LZTR1*-CM contained higher levels of GDF-15, CXCL1 and GM-CSF than painful NEC-CM and painful *SMARCB*1-CM. These findings indicate an association between distinct profiles of secreted cytokines and chemokines in schwannomas of patients with germline PVs in different SWN genes [118]. These authors also investigated the pain response behaviour of mice after CM injection. All CM from painful schwannomas caused an increase in licking and flinching compared to control media. However, only painful *LZTR1*-CM caused a significantly increased acute pain response compared to non-painful *LZTR1*-CM. Furthermore, the increase in pain response after injection of painful *LZTR1*-CM was higher compared to the response after injection of painful *SMARCB1*-CM and NEC-CM.

Pre-treatment of cultured mouse neurons with CM from painful schwannoma cell lines enhanced their responsiveness to noxious TRPV1 and TRPA1 agonists. However, this responsiveness was different when comparing *LZTR1*-CM, *SMARCB1*-CM and NEC-CM. Painful *SMARCB1*-CM and *LZTR1*-CM enhanced the response to low-dose capsaicin more than NEC-CM. Conversely, painful NEC-CM evoked a significantly higher response to low-dose cinnamaldehyde than painful *LZTR1*-CM and *SMARCB1*-CM.

Taken together, CM from painful schwannomas sensitized mice to painful stimuli. The injection of CM from painful schwannomas in mice evoked more acute pain than did CM from non-painful schwannomas of patients with non-*NF2* SWN. Further, the behavioural effects of CM injection were different when comparing CM derived from schwannomas of patients with PVs in different SWN-related genes. Additionally, the cytokine and chemokine content of CMs were different comparing schwannomas derived from patients with different forms of SWN [118].

#### Schwannomas

Schwannomas are the most common tumours in all types of SWN [29, 32]. In patients with non-*NF2*-related SWN, schwannomas of the peripheral nerves have been observed in 81–89% of patients whereas spinal schwannomas have been noted in 74% of these patients [29, 32]. Both peripheral nerve and spinal schwannomas are less common in *NF2*-related SWN than in non-*NF2*-SWN [32] (Table 3). Intra-dermal schwannomas are common in *NF2*-related SWN but very rare in patients with non-*NF2*-related SWN [119] and even considered to be completely absent in non-*NF2*-related SWN [29, 31, 64].

**Table 3:** Frequency of tumours in the different types of schwannomatosis (SWN) after Evans et al. [32]

Tumour type/ location	Non-*NF2*-related SWN^a^	*NF2*-related SWN
Peripheral nerve schwannomas	81%^b^	38.5%
Spinal schwannomas	74%^c^	66%
Trigeminal nerve schwannoma	11%	27%
Lower cranial nerve schwannoma	4.5%	15.5%
Vestibular schwannoma	7–16%^d^	94%
Facial schwannoma	9%	19.5%
Meningioma	4.5%^e^	53%^f^
Ependymoma	0%	19%
Malignant peripheral nerve sheath tumour (MPNST)	Reported^g^	0% without radiation

^a^Non-*NF2*-related SWN affects patients who fulfil the diagnostic criteria for SWN according to [64] (Supplementary Table 1)

^b^Intra-dermal schwannomas are common in patients with *NF2*-related SWN but very rare or even absent in patients with non-*NF2*-related SWN

^c^Spinal schwannomas are significantly more common in patients with *LZTR1*-related SWN (5/5, 100%) than in patients with *SMARCB1*-related SWN (6/15, 40%) according to [104]

^d^Unilateral vestibular schwannomas have so far only been observed in patients with *LZTR1*-related SWN [32, 68, 81, 83, 93, 96]. Histologically confirmed vestibular schwannomas have not been reported in patients with *SMARCB1*-related SWN

^e^Only observed in patients with *SMARCB1*-related SWN, not in patients with *LZTR1*-related SWN

^f^Meningioma is often the first tumour detected in a child with *NF2*-related SWN with an early presentation of symptoms

^g^MPNSTs have been reported in patients with *SMARCB1*-related SWN but not in other types of SWN [34, 38, 40, 54, 102, 140]. Hence the MPNST risk is increased in *SMARCB1*-related SWN but not in other types of SWN without prior irradiation

Bilateral vestibular schwannomas are the hallmark feature of *NF2*-related SWN, and are present in 88% of patients with germline *NF2* PVs older than 30 [79, 83, 120]. Bilateral vestibular schwannomas are an important diagnostic criterion for *NF2*-related SWN [32, 121] and appear to be absent or are at least extremely rare in non-*NF2*-related SWN [32, 65, 122]. So far, only one patient with a germline pathogenic *LZTR1* variant has been reported with bilateral vestibular schwannoma. The clinical presentation of this patient was however atypical and distinct from patients with *NF2*-related SWN as hearing was never lost and the second tumour formed quite late in life at the age of 47 [65]. In contrast to bilateral vestibular schwannomas, unilateral vestibular schwannomas (UV) may occur more often in *LZTR1*-related SWN. UV have been observed in 7–16% of *LZTR1*-related SWN patients [32, 68, 81, 83, 93, 96]. Although there is a single case report of an apparent UV in a family with a germline *SMARCB1* PV, this potential association has not yet been validated [123]. The study of larger cohorts of patients with *SMARCB1*-related SWN imply that vestibular schwannomas do not occur in this group of patients [32, 40, 83, 120, 122]. Remarkably, patients with pathogenic *LZTR1* germline PVs appear to have a significantly higher prevalence of spinal schwannomas as compared to patients with *SMARCB1*-related SWN [104].

#### Segmental schwannomas

About one third of patients with non-*NF2*-related SWN develop segmental schwannomatosis, with schwannomas apparently confined to a body segment such as a limb or several spinal nerve roots [29, 124]. As yet, there is no evidence for a causal relationship between the segmental presentation of SWN and genetic mosaicism of either *SMARCB1* or *LZTR1* PVs. Most of the patients with segmental SWN and a pathogenic variant identified in blood harboured *LZTR1* PVs [124, 125]. However, segmental schwannomas have also been reported in a patient with a *SMARCB1* PV (c.92 A > G, p.Glu31Gly). The patient had intradural extramedullary schwannomas only in a region of the thoracic spine (T9–T12) associated with severe pain. Interestingly, her mother possessed the same germline *SMARCB1* PV but exhibited generalized SWN with multifocal (non-segmental) painless extradural neurogenic tumours in various parts of her body [126]. The observed intrafamilial phenotypic heterogeneity suggests that in addition to the nature of the germline PV, other factors such as the timing of the somatic inactivation of the second allele also determine whether schwannomatosis presents as generalized or segmental disease.

#### Meningiomas

Germline mutations in *SMARCB1* also predispose to the development of cranial meningiomas [32, 37, 55, 127–130]. Meningiomas are observed as single tumours in 4–5% of patients with *SMARCB1*-related SWN [32, 38] occurring predominantly in the anterior falx cerebri [55, 131]. Importantly, germline *SMARCB1* PVs do not appear to be a frequent cause of multiple meningiomas even though some families with multiple meningiomas and *SMARCB1*-related SWN have been reported [127, 128, 132]. It should be noted that meningiomas have not so far been observed in patients with *LZTR1*-related SWN. By contrast, 53% of patients with *NF2*-related SWN develop meningiomas [32, 133], which are among the earliest clinical features to become evident in these patients [100] (Table 3). Taken together, *SMARCB1* germline PVs probably represent an occasional cause of meningioma predisposition [130]. Somatic *SMARCB1* mutations not present in the germline may sometimes occur in sporadic meningiomas but these are essentially rare events [134–137].

#### Leiomyomas

Whether germline *SMARCB1* PVs may also predispose to leiomyomas remains unclear. So far, only one patient with *SMARCB1*-related SWN has been reported with a leiomyoma of the cervix uteri [138]. Remarkably, chromosome 22q deletions are frequent in patients with sporadic uterine leiomyomas [139].

#### Malignancy risk in patients with *SMARCB1*-related SWN

Importantly, patients with *SMARCB1*-related SWN but not those with other types of SWN have an increased risk of malignancy. Although malignant rhabdoid tumours are uncommon in patients with *SMARCB1*-related SWN [29, 32], malignant peripheral nerve sheath tumours (MPNSTs) have been reported to occur in these patients [34, 38, 40, 54, 102, 140] (Table 3). MPNSTs are rare in the general population but they occur at an increased frequency in patients with Neurofibromatosis type 1 (NF1). An estimated 20–50% of patients with MPNSTs have NF1 [141, 142]. The lifetime risk of an MPNST in NF1 is 8–13% [102]. In addition to patients with NF1 or *SMARCB1*-related SWN, MPNSTs also occur with higher frequency in carriers of germline *TP53* mutations. However, MPNSTs are not observed at an increased rate in other tumour predisposition syndromes [102]. In contrast to *SMARCB1*-related SWN, MPNSTs are extremely rare in *NF2*-related SWN and almost never occur in the absence of radiation treatment [102, 143]. Furthermore, in patients with *LZTR1*-related SWN, MPNSTs have not so far been reported.

An association between specific pathogenic *SMARCB1* variants and the occurrence of MPNSTs in schwannomatosis patients has not been identified. It is likely that additional genetic alterations drive malignant transformation of schwannomas in patients with *SMARCB1*-related SWN. In addition to an increased risk of MPNST, a more extended malignancy phenotype may be associated with *SMARCB*1-related SWN. Eelloo et al. [140] reported a 51-year old female patient with a germline pathogenic *SMARCB1* variant and multiple benign and malignant tumours including schwannomas, follicular lymphoma (WHO grade II), neurofibroma, uterine leiomyoma, MPNST and a neuroendocrine carcinoma of the kidney. The patient had a single base-pair deletion in *SMARCB1* exon 1 causing a frameshift (c.38del; Lys13Serfs*3). Pathogenic variants in *SMARCB1* exon 1 are quite frequent in patients with *SMARCB1*-related SWN [40] (Sect. Hypomorphic or semi-functional *SMARCB1* PVs in patients with SWN).

Other malignant tumours observed in patients with *SMARCB1*-related SWN include papillary renal cell carcinoma [144] and leiomyosarcoma [145]. Owing to the increased risk of malignancy in patients with *SMARCB1-*related SWN, it has been recommended that a growing tumour, especially one causing increasingly severe functional impairment, should be immediately investigated for possible malignant transformation [103].

### Rhabdoid tumour predisposition syndrome type 1 (RTPS1)

Approximately 25–35% of patients with malignant rhabdoid tumours (MRTs) carry a germline *SMARCB1* alteration, which defines the Rhabdoid Tumour Predisposition Syndrome type 1 (RTPS1) [12–15, 146, 147]. In rare cases, patients with MRT harbour germline PVs in *SMARCA4*; this causes RTPS2 and is much less common than RTPS1 [15, 148]. Biallelic loss of function of *SMARCB1* drives malignancy in MRTs, which comprise a group of highly aggressive embryonal tumours. MRTs occur predominantly in young children, frequently leading to death within the first few years of life [2–6, 8, 149]. MRTs commonly arise in the central nervous system and are termed atypical teratoid/rhabdoid tumours (AT/RT). Other anatomical sites of MRTs are extracranial including head and neck, paravertebral muscles, liver, bladder, mediastinum, retroperitoneum, extremities, pelvis, heart and kidney [reviewed by 150]. MRTs contain rhabdoid cells, characterized by eccentric vesicular nuclei with prominent nucleoli and glassy eosinophilic, inclusion-like cytoplasmic structures, which are aggregates of intermediate filaments [151]. Loss of nuclear SMARCB1 protein expression is the diagnostic hallmark of AT/RT which make up 1–2% of all CNS tumours in children, primarily affecting infants (> 70%) [reviewed by 9]. They belong to the WHO grade 4 embryonal CNS tumours group [152] and represent 40–50% of CNS tumours occurring during the first year of life [11]. Although AT/RTs develop in a variety of brain regions, the posterior fossa seems to be a frequent location [149, 153]. AT/RTs are highly malignant cancers with substantial clinical heterogeneity, poor prognosis and low overall survival rates [149, 154–160, reviewed by 11]. Even though AT/RTs occur mainly in infants and very young children, they also develop rarely as primary tumours in adults and then predominantly in the sellar region [161–170].

In patients with RTPS1, tumours develop at an earlier age than in patients without germline *SMARCB1* PVs [12, 155, 171]. The very young age of patients with RTPS1 may account for their poorer prognosis compared to patients with somatic *SMARCB1* PVs and AT/RT [12]. However, the association between germline predisposition to MRT and prognosis is controversial. Upadhyaya et al. [172] did not observe an association between germline predisposition by a *SMARCB1* PV and a poor prognosis of AT/RT. However, in contrast to this, other studies confirmed just such an association [53, 56, 173]. Likewise, Frühwald et al. [15] reported that in addition to young age (younger than one year), a germline *SMARCB1* PV is a negative prognostic factor for the survival of patients with AT/RT. Familial penetrance of RTPS1 is approximately 90% by the age of 5 [150]. The median age of diagnosis of a rhabdoid tumour in patients with RTPS1 is between 4 and 7 months. By contrast, the age at diagnosis in sporadic patients with AT/RT is 18–30 months [150]. Synchronous tumours occur in one third of patients with RTPS1, with the kidney as the most common synchronous site [146, 173–176].

#### Co-occurrence of RTPS1 and schwannomatosis in families

An estimated one-third of patients with MRT and germline *SMARCB1* PV are familial cases [13]. In these families, the pathogenic heterozygous *SMARCB1* variant detected in the child with MRT is also present in the blood of one of their parents [13]. Four families have so far been reported, with carriers of germline pathogenic *SMARCB1* variants having either schwannomatosis or MRT [13, 52, 54, 177]. A common pattern observed in these families was that the parent who carried the *SMARCB1* PV had schwannomatosis whereas their offspring had MRT. Importantly, in familial cases of MRT, at least 17 unaffected *SMARCB1* PV carriers have been reported [8, 33, 50–56]. These *SMARCB1* PV carriers were unaffected in the sense that they did not develop MRT or symptomatic schwannomas. However, it is unclear whether these apparently unaffected *SMARCB1* PV carriers had clinical signs of *SMARCB1*-related SWN since they were not investigated by MRI, especially later in life, in order to exclude the occurrence of asymptomatic schwannomas. The incomplete penetrance observed in these families in terms of MRT development is caused by the fact that a narrow developmental window exists during which neural crest cells are sensitive towards the complete loss of the SMARCB1 protein thereby initiating rhabdoid tumour growth [178, 179, reviewed by 180]. If this sensitive period is completed prior to biallelic *SMARCB1* inactivation, MRT may not develop at all (Sect. The time window of *SMARCB1* inactivation in rhabdoid tumour development). However, schwannomatosis is highly likely to occur later in life in these *SMARCB1* PV carriers without MRT since the penetrance of *SMARCB1*-related SWN is high, most likely 100% [103]. This estimate cannot be given more precisely since *SMARCB1*-related SWN is often diagnosed after the age of 30 and can only be confirmed or excluded by comprehensive MRI investigation. It should be noted that the co-occurrence of MRT and *SMARCB1*-related schwannomatosis in the same patient has been observed in long-term survivors of AT/RT thereby substantiating this hypothesis.

#### RTPS1 long-term survivors may develop schwannomas

Patients with RTPS1 have a very poor prognosis due to malignancy in infancy or early childhood. Long-term survival in children with AT/RT is very rare [51, 181, 182]. However, with improved treatment strategies, patients have been reported who survived the childhood MRT. Several of them developed additional primary *SMARCB1*-deficient tumours after being cured of the initial MRT. The tumours observed in AT/RT survivors that developed beyond the age of 5 included extracranial MRT [12, 147], AT/RT [183], MRT of the kidney [184], epitheloid sarcoma [185], schwannoma [52, 147, 186], chrondrosarcoma [187], myoepithelioma and meningioma [51], epitheloid malignant peripheral nerve sheath tumour (epitheloid MPNST) [54, 147], MPNST [188], primitive neuroectodermal tumour (PNET) [5] and adult sellar AT/RT [167]. This indicates that patients with RTPS1 remain at elevated risk for developing *SMARCB1*-deficient tumours after the peak age of MRT in early childhood. Consequently, clinical surveillance of RTPS1 patients beyond the age of 5 is very important [147]. It may be argued that in these MRT long-term survivors, tumour stem cells persist that are vulnerable to a critical second hit, which would drive malignancy later in life.

#### Cribriform neuroepithelial tumour (CRINET)

In addition to rhabdoid tumours such as AT/RT or extracranial MRT, patients with germline *SMARCB1* PVs and RTPS1 appear to be predisposed to rare non-rhabdoid tumours such as cribriform neuroepithelial tumours (CRINETs). CRINETs are rare embryonal CNS tumours mostly diagnosed in children younger than 2.5 years [152, 189, 190]. Only rarely do children older than 2.5 years develop CRINETs [190, 191]. CRINETs also exhibit biallelic *SMARCB1* loss as observed in MRTs [190–193]. In CRINETs, *SMARCB1* loss leads to high tyrosinase expression, strikingly resembling the AT/RT-TYR subgroup based on DNA methylation and gene expression profiles [190] (Sect. AT/RT subgroups). Moreover, CRINETs and AT/RT-TYR both harbour large heterozygous losses of chromosome 22, with accompanying intragenic pathogenic variants of the other *SMARCB1* allele, which is uncommon in other AT/RT subgroups. Nevertheless, CRINETs exhibit distinct histopathological features and a more favourable long-term outcome than tumours of the AT/RT-TYR subgroup [190]. In the majority of patients with CRINET, *SMARCB1* PVs are somatic and accompanied by loss of heterozygosity of the other allele. However, two patients with CRINETs and germline *SMARCB1* PVs have been reported [190] suggesting that CRINETs belong to the spectrum of tumours that may occur in patients with RTPS1.

#### Malignant melanocytic uveal tumour

Another tumour type that may expand the spectrum of RTPS1-associated tumours is melanocytic uveal tumour [194]. These authors reported two cases of aggressive intraocular tumours in two children with germline *SMARCB1* PVs and biallelic *SMARCB1* loss in tumour tissue. The genomic profiles as well as the transcriptome and DNA-methylation profiles of these *SMARCB1*-deficient malignant melanocytic uveal tumours were clearly different from MRT and uveal melanomas [194]. One of the two patients identified by Cyrta et al. [194] was treated at the age of 15 months for a localised AT/RT. After intensive treatment, she achieved complete remission. Surveillance revealed no sign of recurrence until 11 years of age, when she presented with an asymptomatic lesion of the left eye on a systematic follow-up MRI. The lesion initially showed slow growth, but underwent progression at the age of 14 to a malignant melanocytic uveal tumour with complete loss of *SMARCB1* protein expression.

The second patient reported by Cyrta et al. [194] had not developed an MRT during her early years and had no family history of RTPS1. At the age of 23, however, she was diagnosed with a malignant uveal tumour. Blood analysis indicated a *de novo* germline 3.1 Mb deletion on 22q11.2 encompassing 38 genes including *SMARCB1*. The patient did not exhibit dysmorphic features or intellectual disability as observed in some patients with large deletions in the distal 22q11.2 region including *SMARCB1* [156, 195]. Such large deletions in distal 22q11.2 encompassing 2-3-Mb are not frequent in patients with RTPS1 and only 10 patients with deletions of this type and AT/RT have been reported to date [196–201]. In the tumour tissue of the patient with the 3.1-Mb distal deletion in 22q11.2, complete loss of *SMARCB1* expression was observed [194]. Thus, *SMARCB1*-deficient malignant melanocytic intraocular tumours would appear to be part of the spectrum of RTPS1-associated tumours.

### Neurodevelopmental disorders caused by germline *SMARCB1* PVs

#### Coffin-Siris syndrome (CSS)

Germline PVs in *SMARCB1* cause the clinically and genetically heterogeneous Coffin-Siris syndrome (CSS, MIM #135900) [45–49, 202–209]. It is estimated that 7% of all patients with CSS carry a germline pathogenic *SMARCB1* variant. PVs in other genes encoding members of the BAF complex also cause CSS: *ARID1B* (MIM #614556), *ARID1A* (MIM #603024), *SMARCA4* (MIM #603254) and *SMARCE1* (MIM #603111) [45, 210, 211]. Approximately 60% of all patients with CSS harbour PVs in genes encoding members of the BAF chromatin remodelling complex [46, 212–214].

Pathogenic variants in *SMARCB1* generally cause a very severe CSS phenotype with global developmental delay and in most instances, severe intellectual disability [47, 48, 213, 214]. CNS abnormalities (mainly agenesis of the corpus callosum), seizures and absence of speech are common features in *SMARCB1-*related CSS [213–215]. Cardiovascular defects (septal defects, pulmonal artery stenosis, and/or dextrocardia), gastrointestinal problems (mainly gastro-esophageal reflux or pyloric stenosis) and genitourinary complications are also frequent in patients with CSS caused by germline *SMARCB1* PVs. Feeding difficulties, postnatal growth retardation, sparse scalp hair, severe scoliosis and hypoplastic 5th fingers/toes and hypoplastic 5th fingernails/toenails are also common in these patients [47, 48, 213, 214]. Patients with *SMARCB1*-related CSS also have a marked progressive coarseness of the face with dysmorphic facial features including hypertelorism, thick eyebrows, a depressed and broad nasal bridge, anteverted nares and a large mouth with macroglossia [213, 214, reviewed by 216].

#### Intellectual disability with choroid plexus hyperplasia (ID-CPH)

A recurrent missense pathogenic variant in the N-terminal part of *SMARCB1* causes severe intellectual disability and choroid plexus hyperplasia with resultant hydrocephalus, termed ID-CPH [217]. The pathogenic *de novo* missense *SMARCB1* variant (c.110G > A; p.Arg37His) responsible was first identified in an individual with a clinical presentation overlapping with Kleefstra syndrome (KS) (MIM #610253) [218] and subsequently in an additional three unrelated individuals. The four patients showed a similar clinical phenotype including severe intellectual disability, hydrocephalus due to choroid plexus hyperplasia, various congenital anomalies, severe feeding difficulties, anemia, sleep apnea and ophthalmological problems. Some similarities were noted between individuals with CSS and the four patients with ID-CPH such as severe intellectual deficits, congenital heart defects, kidney anomalies, and feeding difficulties. However, other features frequently observed in patients with CSS caused by *SMARCB1* PVs including impaired growth, microcephaly, fifth digit anomalies, dystrophic scoliosis and epilepsy were absent in the patients with ID-CPH.

The most distinctive phenotypic feature associated with the p.Arg37His variant in patients with ID-CPH was the enlargement of the central cerebrospinal fluid spaces, often leading to high-pressure hydrocephalus associated with choroid plexus hyperplasia and overproduction of cerebrospinal fluid [217]. This clinical feature has not been observed in patients with CSS implying that ID-CPH and CSS are different clinical entities within the spectrum of syndromes associated with PVs in *SMARCB1* [217].

#### DOORS syndrome

DOORS syndrome (Deafness, Onychodystrophy, Osteodystrophy, mental Retardation, Seizures) is characterized mainly by sensorineural deafness, shortened terminal phalanges with small nails on hands and feet, increased urinary 2-oxoglutaric acid excretion, intellectual deficiency and seizures [219]. Pathogenic variants within the *TBC1D24* gene (MIM #613577) are observed in approximately 50% of the patients exhibiting all the aforementioned clinical features. The genetic analysis of 32 families (36 patients) with DOORS syndrome indicated *TBC1D24* PVs in 13 individuals from 10 families [219]. Subsequent whole exome sequencing in patients from the cohort without *TBC1D24* PVs indicated the *de novo SMARCB1* PV (c.1130G > A; p.Arg377His) in two unrelated patients. Remarkably, this PV is also known to cause Coffin-Siris syndrome (Table 4, Sect. Molecular pathogenesis of Coffin-Siris syndrome (CSS)). In contrast to the other patients in this cohort with DOORS syndrome, the patients with *SMARCB1* PVs did not have seizures and 2-oxoglutaric aciduria. They also exhibited coarse facial features, 5th finger hypoplasia and cardiovascular malformations which occur more frequently in patients with CSS than in those with *TBC1D24-*deficient DOORS syndrome. The differential diagnosis was however impaired by the very young age of both patients with the pathogenic *SMARCB1* variant - one even died during the neonatal period [219]. In view of the overlap of clinical symptoms between these two patients with DOORS syndrome and those with CSS [46–48, 213, 214], it might be possible that a small subgroup of patients with atypical CSS may also exhibit symptoms associated with DOORS syndrome.

**Table 4:** *SMARCB1* pathogenic variants identified in 35 patients with Coffin-Siris syndrome (CSS)

Pathogenic variant (PV)	Exon	PV type	Amino acid change	Reference	Patient ID	Age at diagnosis	Gender	Inheritance
c.31G > A	1	missense	p.Gly11Arg	204	88_S3	1–5 y	F	*De novo*
c.31G > A	1	missense	p.Gly11Arg	209	ID-28	ns	ns	*De novo*
c.806 A > G	7	missense	p.His269Arg	205	156	ns	ns	*De novo*
c.1052dup	8	frameshift	p.Leu352Thrfs*9	205	235	ns	ns	*De novo*
c.1066_1067del	8	frameshift	p.Leu356Aspfs*4	208	Fetus 1	ns	ns	*De novo*
c.1087 A > G	8	missense	p.Lys363Glu	205	180	ns	ns	*De novo*
c.1089G > T	8	missense	p.Lys363Asn	48	43	ns	ns	*De novo*
c.1091 A > C	8	missense	p.Lys364Thr	207	1	33 y	M	paternal
c.1091 A > C	8	missense	p.Lys364Thr	207	2	36 y	F	paternal
c.1091_1093del	8	ifd	p.Lys364del	45	4	21 y	F	*De novo*
c.1091_1093del	8	ifd	p.Lys364del	45	21	7 y	F	ns
c.1091_1093del	8	ifd	p.Lys364del	45	22	2 y	M	ns
c.1091_1093del	8	ifd	p.Lys364del	46	29	ns	ns	*De novo*
c.1091_1093del	8	ifd	p.Lys364del	46	37	ns	ns	*De novo*
c.1091_1093del	8	ifd	p.Lys364del	46	48	ns	ns	*De novo*
c.1091_1093del	8	ifd	p.Lys364del	48	5	ns	ns	*De novo*
c.1091_1093del	8	ifd	p.Lys364del	48	18	ns	ns	ns
c.1091_1093del	8	ifd	p.Lys364del	48	37	ns	ns	*De novo*
c.1091_1093del	8	ifd	p.Lys364del	392	ns	28 y	F	*De novo*
c.1091_1093del	8	ifd	p.Lys364del	205	174	ns	ns	*De novo*
c.1091_1093del	8	ifd	p.Lys364del	205	136	ns	ns	*De novo*
c.1091_1093del	8	ifd	p.Lys364del	393	10	7.5y	F	*De novo*
c.1091_1093del	8	ifd	p.Lys364del	209	ID-29	ns	ns	*De novo*
c.1096 C > T	8	missense	p.Arg366Cys	49	K2588	ns	ns	*De novo*
c.1096 C > T	8	missense	p.Arg366Cys	202	MR2788	11 months	M	*De novo*
c.1096 C > T	8	missense	p.Arg366Cys	206	4	13 y	F	*De novo*
c.1096 C > G	8	missense	p.Arg366Gly	205	88	ns	ns	*De novo*
c.1107 C > G	8	missense	pAsp369Glu	394	43	ns	ns	*De novo*
c.1113 C > G	8	missense	p.Asn371Lys	203	8	7 months	F	*De novo*
c.1121G > A	9	missense	p.Arg374Gln	282	ns	26 y	M	*De novo*
c.1121G > A	9	missense	p.Arg374Gln	49	K2426	4 months	M	*De novo*
c.1121G > A	9	missense	p.Arg374Gln	205	230	ns	ns	*De novo*
c.1130G > A	9	missense	p.Arg377His	45	11	7 y	F	*De novo*
c.1130G > A	9	missense	p.Arg377His	394	44	ns	ns	*De novo*
partial deletion	8 + 9	9 kb deletion	from intron 8 extending into the flanking *DERL3* gene	205	076	ns	ns	*De novo*

ifd: in-frame deletion; ns: not specified; y: year(s); M: male, F: female

### Distal 22q11.2 deletion syndrome

**Fig. 2:**
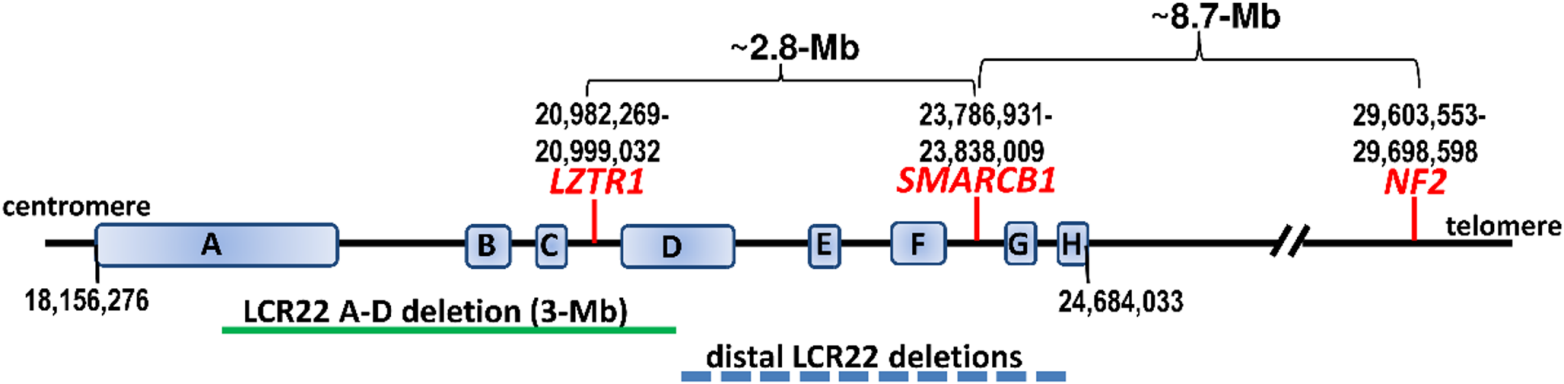
Schematic representation of the proximal chromosome 22q region (22q11.2) indicating the locations of the *LZTR1* and *SMARCB1* genes and the low copy repeats 22 (LCR22 A-H). The nucleotide numbering is given according to GRCh38.p14/hg38. The genomic positions of the LCRs are: LCR22-A: 18,156,276 − 19,035,473; LCR22-B: 20,141,014–20,377,631; LCR22-C: 20,667,276 − 20,738,272; LCR22-D: 21,009,379 − 21,565,091; LCR22-E: 22,617,530 − 22,707,515; LCR22-F: 23,307,813 − 23,477,813; LCR22-G: 24,234,032 − 24,304,032; LCR22-H: 24,599,033 − 24,684,063. The *NF2* gene is located telomeric to *LZTR1* and *SMARCB*1 at 22q12.1

Chromosome 22q11.2 contains regions of multiple low copy repeat (LCR) sequences that mediate non-allelic homologous recombination (NAHR) and predispose to pathogenic deletions and duplications [reviewed by 220]. Eight LCRs located at chromosome 22q11.2, designated as LCR22 A–H, have been identified [220] (Fig. 2). Patients with germline distal deletions in 22q11.2, encompassing sub-bands 22q11.22-q11.23 and mediated by NAHR between LCR22 D-H, have an increased risk of MRT (Fig. 2). Importantly, these distal deletions (type III) [195] encompass the *SMARCB1* gene which explains the predisposition to rhabdoid tumours in these patients [12, 13, 199]. So far, 17 patients with type III deletions have been reported and many of them exhibit congenital anomalies including dysmorphic features, cardiac defects, developmental delay and microcephaly [12, 195–199, 221, 222]. A proportion of these patients also exhibit intellectual deficits, language delay and psychiatric or behavioural problems suggesting that *SMARCB1* deficiencies are not only responsible for the increased MRT risk in these patients but also for disturbances during neurodevelopment. In addition to large deletions in the distal 22q11.2 region, other structural rearrangements such as ring-chromosome 22 also predispose to the development of AT/RT [223].

## Molecular pathogenesis of *SMARCB1*-related schwannomatosis and other *SMARCB1*-associated phenotypes

### SMARCB1

*SMARCB1* (SWI/SNF related, matrix associated, actin-dependent regulator of chromatin, subfamily b, member 1) encodes a core subunit of the BAF chromatin-remodelling complex responsible for regulating gene expression and development by positioning/remodelling nucleosomes at genomic regulatory regions in an ATP-dependent manner [224, 225]. BAF complex activity is essential for pluripotency in embryonic stem cells, regulating accessibility and transcription at promoters, enhancers and pluripotency factor binding sites [226–229, reviewed by 230]. *SMARCB1* is involved in cell growth regulation and cytoskeleton reorganization [231–235]. *SMARCB1* loss in MRT cells alters the translation efficiency of specific mRNAs [236]. *SMARCB1* is ubiquitously expressed in the nuclei of all normal cells [237] and acts as a tumour suppressor in pediatric AT/RT and extracranial MRTs. In adult tumours, *SMARCB1* may have a more multifaceted, even oncogenic role [238, 239].

#### Structure of the *SMARCB1* gene

The *SMARCB1* gene is located on chromosome 22q11.23, approximately 5.9-Mb centromeric to the *NF2* gene. *SMARCB1* encompasses nine exons but a complex pattern of splice variants of *SMARCB1* exists with at least eight mRNA isoforms [240]. The most common ones are isoform 1 with a full-length exon 2 (exon 2 L, 139 bp), and isoform 2, which contains a shorter exon 2 lacking the last 27 nucleotides at its 3′ end (exon 2 S, 112 bp) [240, 241]. The other six isoforms represent a small proportion of all *SMARCB1* transcripts and their functional significance remains unclear. Intriguingly, there is some functional redundancy between the two major *SMARCB1* isoforms 1 and 2 [37]. Indeed, compensatory expression is observed, such that knockdown of either isoform alone has no effect on cell survival [242]. Pronounced functional differences between the major isoforms have not been reported in comparative studies [243, 244]. This is important because pathogenic variants that affect only one *SMARCB1* isoform may be compensated, or partially compensated for, by the other isoform, which may result in residual *SMARCB1* function. Such hypomorphic pathogenic *SMARCB1* variants may explain why patients with these variants and *SMARCB*1-related SWN exhibit benign schwannomas during adulthood but usually do not develop the highly malignant pediatric AT/RT characterized by the complete loss-of-function of *SMARCB*1 (Sect. Hypomorphic or semi-functional *SMARCB1* PVs in patients with SWN).

#### SMARCB1 as a core subunit of BAF and PBAF

The SMARCB1 protein (also termed BAF47) is required for widespread BAF complex-mediated activation of enhancers and bivalent promoters [245–247]. As a core subunit, SMARCB1 is included in two of the BAF complex family members, the canonical BAF complex (cBAF or simply BAF) [248] and the polybromo-associated BAF complex (PBAF) [249]. However, SMARCB1 is not included in the non-canonical BAF complexes [250–252]. The BAF subcomplexes contain 12–15 subunits encoded by 29 genes [reviewed by 253]. As the catalytic subunit, BAF and PBAF subcomplexes each contain a single ATPase (encoded by either *SMARCA2* or *SMARCA4*) as well as three core subunits, including SMARCB1, and additional accessory subunits [reviewed by 254]. The specific composition of the subunits varies between different tissues. BAF complexes use energy derived from ATP hydrolysis to destabilize histone-DNA interactions and alter nucleosome positions, thereby increasing the accessibility of DNA-binding factors to their genomic target sites and activating gene expression. A model of BAF complex-dinucleosome interaction implies that one nucleosome occupies a large pocket on the surface of the BAF complex and stimulates its ATPase-driven DNA translocase activity. The nucleosome in the pocket retains all of its histones, although its structure is altered, while a neighbouring nucleosome in the path of the mobilized nucleosome-BAF complex is evicted from the DNA [255, 256].

Within the BAF complex, SMARCB1 acts as an anchor that binds to the nucleosome acidic patch via its highly basic C-terminal alpha helical domain, whereas the ATPase subunit SMARCA2 or SMARCA4 binds to the opposing side of the nucleosome [257–260]. With the nucleosome held on both sides, the ATPase subunit is able to slide DNA along the nucleosome [20].

As determined by the analysis of *SMARCB1* re-expression in *SMARCB1*-deficient MRT cell lines, the BAF complex facilitates the acetylation of histone 3 lysine 27 (H3K27) through its interaction with histone acetyltransferases. This leads to increased gene and enhancer activity and antagonizes the effect of the polycomb repressive complex (PRC2) which is responsible for the trimethylation of H3K27 [22, 245, 246, 261, 262] (see Sect. BAF and Polycomb complex antagonism).

The three BAF subcomplexes (cBAF, PBAF and ncBAF) have distinct functions determined by the incorporation of complex-specific subunits [250, 263]. For example, the BAF subcomplexes play different roles in macrophage responses to bacterial endotoxins, regulating chromatin accessibility and enhancer activation, thereby influencing the expression of inflammatory response genes [264]. Further, PBAF (but not cBAF) is essential to the maintenance of genomic integrity during mitosis. Additionally, PBAF but not cBAF plays an important role during DNA-damage-induced transcriptional repression involving the polycomb repressive complexes 1 and 2 [265] and the ubiquitination of proliferating cell nuclear antigen (PCNA) induced upon DNA damage [266].

Emerging evidence also points to distinct localization patterns for each BAF subcomplex. The BAF complex preferentially binds to active distal enhancers, which are located some distance away from the gene they regulate [246]. By contrast, PBAF is enriched at proximal promoter regions [246, 267, reviewed by 20].

Some of the BAF/PBAF subunits are tissue- or cell-specific and the combinatorial assembly of tissue-specific complexes plays an important role during cell lineage acquisition [reviewed by 254]. During differentiation, the BAF-complex composition changes in a tightly regulated manner. Many of the resulting complexes are responsible for tissue-specific regulation of neural development and function, heart development, muscle development or embryonic stem cell pluripotency [reviewed by 268]. The timely expression of specific BAF subunits directs stem cell fate in neurogenesis as well as in skeletal myogenesis [reviewed by 216]. The localization of BAF complexes coincides with the major fate-determining factors of the cell lineage [reviewed by 253]. Indeed, embryonic stem cells have a BAF complex comprised of a specialized subunit composition not found in other cell types (designated as esBAF) which binds strongly to pluripotency factors [226]. During neurogenesis, the esBAF complex undergoes sequential developmental changes in subunit composition and is reconstituted in neural stem cells to the neuronal progenitor BAF (npBAF) complex in order to confer multipotency, while maintaining proliferative ability. As neural development moves on and neural progenitors differentiate into neurons, the npBAF complex switches subunits to form the neuron-specific nBAF complex found only in postmitotic neurons and required for dendritic morphogenesis [263, 269, 270]. SMARCB1 is an essential subunit within these BAF complexes, which play important roles during neural development, neural tissue specification, neuronal migration, maturation and dendritic morphogenesis [reviewed by 254]. Pathogenic variants in genes encoding the BAF complex subunits, including *SMARCB1*, are responsible for different neurodevelopmental disorders. This serves to emphasize the essential role of BAF complex activity during neural development (Sect. Molecular pathogenesis of neurodevelopmental disorders caused by germline *SMARCB1* PVs).

#### Domains of the SMARCB1 protein

**Fig. 3:**
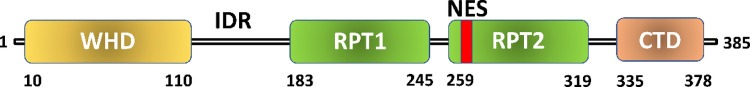
Structure of the SMARCB1 protein: WHD: winged helix domain; IDR: intrinsically disordered region; RPT1 and RPT2: tandem repeat domains; NES: nuclear export signal; CTD: C-terminal coiled-coil domain. The numbers indicate the amino-acid positions

SMARCB1 is a 47 kDa nuclear protein encompassing 385 amino acids. Structurally, it has four distinct domains and a key region designated as an intrinsically disordered region (IDR) (Fig. 3).

##### Winged-helix DNA-binding domain (WHD)

The N-terminal WHD of the SMARCB1 protein exhibits structural similarity to other winged-helix domains found in many different DNA-binding proteins. Nuclear magnetic resonance (NMR) studies using recombinant SMARCB1 WHD expression indicated its ability to bind to double stranded DNA [271]. Within the canonical BAF and the PBAF complex, the WHD appears to adopt different conformations because in BAF, the WHD is located distal to the nucleosome, whereas in PBAF, the WHD is located proximal to the nucleosome [260, 272, 273]. The cryo-EM structure of the nucleosome-bound BAF-complex modelled by He et al. [260] indicated that the SMARCB1-WHD binds the ARM (armadillo repeat) domain of ARID1A (T-rich interactive domain-containing protein 1 A) and is located more than 40Å away from nucleosomal DNA implying a role independent of its DNA-binding ability. Furthermore, the WHD is in close proximity to the actin-related protein (ARP) module, suggesting a role in regulating intermodular interactions [260].

##### Intrinsically disordered region (IDR)

SMARCB1 consists of two globular functional domains that are connected by an intrinsically disordered region (IDR). IDRs enable conformation flexibility and adaptability within proteins in order to facilitate regulation via post-translational modifications, scaffolding and recruitment of transient binding partners, and complex assembly [274]. Within the IDR is a cluster of loss-of-function intolerant residues (aa122, aa123 and aa125) [273]. Missense PVs affecting these residues may impair the flexibility of the SMARCB1 protein within the BAF complex [273].

##### Repeat domains (RPT1 and RPT2)

The tandem repeat domains RPT1 and RPT2 contain two highly conserved imperfect repeat regions encompassing approximately 60 amino acids [275]. RPT1 is necessary for MYC and HIV-1 integrase binding to SMARCB1 [1, 276, 277]. RPT2 interacts with exportin 1 (XPO1) via a nuclear export signal [278]. Normally, this nuclear export signal (NES-residues 259–276) is masked by the C-terminal domain (residues 319–385) of SMARCB1 [278]. Introduction of a truncated SMARCB1 lacking the C-terminal domain into an MRT cell line led to the cytoplasmic localization of SMARCB1. This cytoplasmic localization is dependent upon exportin 1 (XPO1), which directly interacts with the NES sequence of SMARCB1 potentiating its cytoplasmic localization. Importantly, the BAF complex exerts its biological function only in the nucleus. Thus, the cytoplasmic localization of SMARCB1 eliminates its tumour suppressor function. In AT/RTs with pathogenic variants in the C-terminal region of SMARCB1, cytoplasmic SMARCB1 staining is highly enriched in the absence of any nuclear staining of SMARCB1 [279]. Some 19% of all AT/RTs exhibited a cytoplasmic localization for C-terminally mutated SMARCB1 [279]. By contrast, the majority of AT/RT and extracranial MRT exhibit a complete loss of SMARCB1 protein expression [280]. Aberrant cytoplasmic deposition of mutant SMARCB1 protein is frequent only in some AT/RT, specifically in the subgroup with the AT/RT-TYR molecular signature [281] (see Sect. AT/RT subgroups).

##### C-terminal coiled-coil domain (CTD)

The C-terminal coiled-coil domain (CTD) of SMARCB1 contains an alpha-helical region of densely packed basic amino acids (aa 357–377), which physically interact with the nucleosome acidic patch opposite the ATPase catalytic subunit within the BAF complex [258]. Importantly, pathogenic variants of single amino acid residues within the highly basic SMARCB1 alpha helix of the CTD disrupt nucleosome binding and reduce the remodelling efficiency of the BAF complex [258]. Interestingly, these C-terminal mutations have little effect on global BAF localization, suggesting that the specific interaction with nucleosomes is not critical for the binding of BAF complexes to their target genes. PVs in the highly basic *SMARCB1* C-terminal alpha-helical region cause Coffin-Siris syndrome, a neurodevelopmental disorder associated with severe intellectual disability [48, 213, 214] (Sect. Molecular pathogenesis of Coffin-Siris syndrome (CSS)). However, pathogenic variants in the CTD have also been identified in different types of tumour including meningiomas, adenocarcinomas and schwannomas [134, 282, 283].

#### SMARCB1-containing BAF complexes regulate enhancers

SMARCB1 is critical for genome-wide BAF complex binding to enhancers as well as for enhancer activation [245–247, 284]. The reintroduction of *SMARCB1* into *SMARCB1*-deficient MRT cell lines increases BAF localization, particularly at distal enhancers, and promotes active enhancer histone modification marks facilitating gene expression [245–247]. SMARCB1 protein deficiency in MRT cells destabilizes the association of BAF complexes on chromatin, without drastically impairing complex stability or assembly [22, 246, 250]. However, conflicting results have been reported concerning BAF complex stability in the absence of SMARCB1 since some studies have suggested dissociation of the complex due to SMARCB1 loss [247, 285]. Nevertheless, accumulating evidence is suggestive of BAF complex stability in the absence of SMARCB1 [246, reviewed by 20].

In *SMARCB1*-deficient MRT cell lines, re-expression of *SMARCB1* resulted in widespread recruitment of the BAF complex to previously unoccupied enhancers, the activation of these enhancers and the resolution of bivalency at promoters towards an active state [245–247]. This certainly holds true for typical enhancers, but the activity of SMARCB1 at super-enhancers is as yet unclear. Super-enhancers comprise clusters of highly active enhancers and are master regulators of cell identity [reviewed by 286, 287]. Although Nakayama et al. [246] reported significant enhancer activation upon SMARCB1 re-expression in MRT cells at both typical enhancers and super-enhancers, Wang et al. [247] observed that SMARCB1 was dispensable for super-enhancer activation. The results of Alver et al. [245] suggested that the BAF complex within intact SMARCB1 is a major regulator of typical distal enhancer and lineage-specific enhancer activity. However, super-enhancers appeared to be refractory to SMARCB1 loss and less dependent upon BAF complex activation [245]. Further analyses are necessary in order to clarify this organization.

In contrast to MRT cells, which exhibit genome-wide loss of enhancer activity upon *SMARCB1* loss [246, 247, 284], *SMARCB1* knockdown in human embryonic stem cells (hESCs) resulted in widespread transcriptional upregulation and increased expression of bivalent genes [228]. Thus, in differentiating hESCs, the SMARCB1 protein acts as a transcriptional repressor particularly of bivalent genes. Langer et al. [228] also showed that SMARCB1 is essential for hESC super-enhancer silencing during neural differentiation thereby enabling the pluripotent cells to differentiate along this lineage. Loss of SMARCB1 activity in hESCs inhibited neural induction during differentiation assays, a finding which is consistent with its role as a tumour suppressor in the central nervous system [228, 229]. Taken together, SMARCB1 would appear to have differential effects on enhancer and super-enhancer accessibility in a stage- and lineage-specific manner.

During cellular differentiation, regulatory regions such as enhancers become activated by chromatin opening and binding of pluripotency and pioneer transcription factors that confer locus specificity [288, reviewed by 289]. Current models that serve to explain the relationship between chromatin accessibility and transcription include that transcription factors recruit nucleosome remodelers such as the BAF complexes to evict nucleosomes and to facilitate RNA polymerase II (RNAPII) binding. As concluded from murine embryonic stem cell models, RNAPII promoter-proximal pausing promotes BAF occupancy and ATP-dependent nucleosome remodelling, which leads to nucleosome removal and increased DNA accessibility [290]. However, effective chromatin remodelling occurs only at active regulatory regions where simultaneous binding of DNA-sequence-specific transcription factors drives nucleosome eviction [reviewed by 291]. Among these are transcription factors from the Activating Protein 1 (AP-1) family, which, together with lineage-specific transcription factors, bind to nucleosome-occluded enhancers and recruit the BAF complex to induce nucleosome remodelling and establish an accessible chromatin conformation [292, 293]. SMARCB1 inactivation in MRTs results in genome-wide loss of enhancer activity important for normal development [246, 247, 284]. Furthermore, SMARCB1 protein deficiency also impairs the association of lineage-specific transcription factors with enhancers [294]. Re-expression of *SMARCB1* in AT/RT cell lines indicated that BAF complexes with active SMARCB1 subunits are necessary to determine the epigenetic regulatory roles of lineage-specific transcription factors [294]. The AP-1 family of transcription factors plays a central role in this process. Loss of SMARCB1 in a subgroup of AT/RT (AT/RT-MYC, see Sect. AT/RT subgroups) has been shown to lead to the specific loss of expression of the AP-1 subunit c-JUN, which normally organises the expression of lineage-specific transcription factors [294]. Importantly, in melanoma cellular models, loss of c-JUN or other members of the AP-1 transcription factor network is associated with a poorly differentiated state [295]. Thus, the cooperativity between the BAF complex and lineage-specific transcription factors indicates that both are important regulators of cellular identity [294].

Differential regulation by BAF and PBAF complexes has been observed at enhancers and promoters, respectively, suggesting distinct functions of each complex that are perturbed upon SMARCB1 loss in MRT cells [246, 296]. By contrast, both BAF and PBAF complexes are important in activating bivalent promoters during development. Upon SMARCB1 loss-of-function, this process is significantly impaired, resulting in the repression of key tumour suppressor and lineage-specific differentiation genes [246] (see Section BAF and Polycomb complex antagonism).

### Epigenetic and transcriptome changes in rhabdoid tumours

Much of our knowledge about the molecular and pathogenetic consequences of biallelic SMARCB1 protein loss-of-function derives from the analysis of MRT and AT/RT tissue and cell lines. Indeed, biallelic *SMARCB1* inactivation is prevalent in these tumours; other frequently recurring genomic changes including deletions, duplications and pathogenic variants are not observed [10, 24, 26, 297]. Remarkably, rhabdoid tumours are among the tumours with the lowest mutational burden [24, 298, 299]. Despite this lack of genetic heterogeneity, AT/RT exhibit massive changes of their epigenome as is evident from the depletion of H3K27 trimethylation and H3K27 acetylation marks associated with a quiescent genomic state [284, 300, 301].

#### AT/RT subgroups

AT/RTs exhibit heterogeneity in terms of their DNA methylation signatures associated with specific gene expression profiles that may be used to classify AT/RTs into three different subgroups distinguishable by their epigenetic and transcriptome signatures [24, 26, 302–304]. At least three distinct AT/RT molecular subgroups exist: AT/RT-SHH, AT/RT-TYR and AT/RT-MYC [24, 26, 281, reviewed by 305]. The AT/RT-TYR subgroup was named after the enzyme tyrosinase, which is overexpressed in AT/RT-TYR cases, but not in the other AT/RT subgroups, suggesting that tyrosinase immunohistochemistry is a well-suited diagnostic marker for AT/RT-TYR cases [306]. The AT/RT-SHH subgroup displays overexpression of both sonic hedgehog (SHH) and Notch pathway members. Both pathways are conserved key regulators of development [reviewed by 307, 308]. Protein expression of achaete-scute homolog 1 (ASCL1), a neuronal differentiation transcription factor, has been suggested as an immunohistochemical marker for this subgroup [25, 309]. The AT/RT-MYC subgroup exhibits elevated expression of the *MYC* oncogene (MIM #190080) as opposed to the *MYCN* oncogene (MIM #164840), which is overexpressed in the AT/RT-SHH subgroup.

Even if a similar and low mutational burden is common to all AT/RT subgroups, they exhibit substantial differences in their epigenetic profiles. The AT/RT-TYR subgroup, and to a lesser extent the AT/RT-SHH subgroup, exhibit global and promoter hypermethylation comparable to other pediatric brain tumours and normal pediatric brain tissue [24, 304]. By contrast, tumours of the AT/RT-MYC subgroup are characterized by a hypomethylated signature as compared with normal pediatric brain samples [24, 304]. Further, the AT/RT subgroups also exhibit differences in *SMARCB1* mutation patterns, clinical features including patient age, tumour location and neuroradiological imaging results [24, 281, 310]. Several studies have also indicated higher immune cell infiltration in AT/RT-MYC and AT/RT-TYR than in AT/RT-SHH [304, 311, 312].

An association between the AT/RT subgroup, age of the patient and survival has been observed. AT/RT-SHH and AT/RT-MYC DNA-methylation signatures as well as age younger than one year are all negative prognostic factors (5-year overall survival rate: 0%) [15]. By contrast, patients with tumours of the AT/RT-TYR subgroup who were older than one year had a much better prognosis and a 5-year overall survival rate of ~ 70% [15]. Likewise, Upadhyaya et al. [172] observed that infants with tumours in the AT/RT-TYR subgroup had the highest survival rate.

##### Cell-of-origin of AT/RT subgroups

The molecular diversity among the AT/RT subgroups is likely to be associated with a different cell-of-origin. Increasing evidence suggests that AT/RT-SHH derive from neural progenitor cells [281, 313, 314]. Similarities between the DNA methylation and gene expression profiles between extracranial MRT and tumours of the AT/RT-MYC subgroup are suggestive of common dysregulated developmental programs and that they arise from a neural crest-derived lineage shared with Schwann cells and blocked on their way to mesenchymal differentiation [175, 178, 179, 304, 315]. By contrast, the genetically engineered mouse models and single cell transcriptome analyses performed by Graf et al. [316] suggested that AT/RT-MYC as well as extracranial MRTs with the AT/RT-MYC expression profile originate from fetal primordial germ cells (PGCs). *Smarcb1* loss in murine PGCs may cause reversal of germ cell specification, misguided migration to various body locations and finally tumorigenesis [316]. However, neural crest cells and PGCs represent different developmental lineages [317]. Thus, the origin of AT/RT-MYC remains unclear, although the neural crest-derived hypothesis has received the most support [reviewed by 305].

The cellular origin of tumours with the AT/RT-TYR signature also remains unresolved. Although the role of overexpression of tyrosinase in AT/RT tumorigenesis remains to be established, it is remarkable that several other components of the melanosomal pathway are also upregulated in the AT/RT-TYR subgroup, which may indicate a neural crest or neuroectodermal origin for this AT/RT subgroup [281, 318].

It remains to be determined if the AT/RT subgroup-specific characteristics are caused by different cells-of-origin [reviewed by 305]. Organoid and mouse models have indicated a specific early developmental time frame (E6–10) during which SMARCB1 loss-of-function leads to the formation of malignant rhabdoid tumours (see Sect. The time window of *SMARCB1* inactivation in rhabdoid tumour development). Consequently, the pool of potential cells of origin has been narrowed down to early embryonic development [178, 179, 314, 316, 319]. Nevertheless, the cell-of-origin of AT/RTs is still a matter of intensive investigation. Terada et al. [320] established a xenograft model of AT/RT using human *SMARCB1*-deficient pluripotent stem cell-derived neural progenitor-like cells (NPLCs). They observed that that the AT/RT cells-of-origin are undifferentiated cells at a very early developmental stage, before their differentiation into neural progenitor cells. Their analysis also showed that SMARCB1^−/−^ cells are still able to differentiate into neural progenitor cells (NPCs). Importantly, the subsequent neuronal differentiation of NPCs is blocked due to SMARCB1 protein loss-of-function. These findings accord with those of Carmel-Gross et al. [229] who showed that the complete loss-of-function of SMARCB1 in human embryonic stem cells does not impair their capacity to differentiate in vitro and in vivo into NPCs. However, in similar vein to the findings of Terada et al. [320], SMARCB1 deficiency impairs the neuronal differentiation of NPCs. Various other studies have suggested that AT/RT derive from NPCs, in particular those AT/RTs with the AT/RT-SHH molecular signature [281, 313, 314, 319].

It is most likely that cell-of-origin is only one of several important factors during AT/RT development. It has been suggested that AT/RT development may follow a “three-hit-model” which requires that the differentiation stage, cell-of-origin and the type of *SMARCB*1 inactivation (intragenic pathogenic variant, broad deletion and chromosome 22 loss in different combinations) should combine in such a way as to induce tumour development [305].

#### Blocked neural differentiation in AT/RTs

DNA hypermethylation in AT/RT disrupts the binding of transcription factors to DNA which impairs the expression of genes involved in neural differentiation [284, 321]. Importantly, AT/RTs exhibit DNA hypermethylation in the regulatory regions of pioneer transcription factors such as Neurogenin 1 (NEUROG1) and Neuronal differentiation 1 (NEUROD1) [314, 321]. These transcription factors are among the master regulators of neurogenesis and neural differentiation [322-324, reviewed by 289] and the blockage of neural differentiation is causally associated with AT/RT tumorigenesis [313, 319]. Thus, DNA hypermethylation in AT/RTs perturbs neural differentiation which drives malignancy [reviewed by 305]. Indeed, disturbances in cellular differentiation that result in the unlocking of phenotypic plasticity are among the hallmarks of cancer [325]. Halted neural differentiation in AT/RT by loss of BAF complex function is in line with the important role of functional BAF chromatin remodelling and the targeted opening of chromatin during neural development [254]. Murine embryonic stem cell models have indicated that *Smarcb1* plays a crucial role in the development of the nervous system [327, 326, reviewed by 44]. An inducible SMARCB1 loss-of-function system in human induced pluripotent stem cells (iPSCs) has shown that SMARCB1 loss during neuronal differentiation leads to a failure in maturation causing resistance to terminal differentiation [319]. In another tumour model using human *SMARCB1*-deficient pluripotent stem cell-derived neural progenitor-like cells (NPLCs), brain tumours could be induced after the NPLCs were transplanted into the mouse brain [320]. Activation of an embryonic stem cell (ESC)-like signature was associated with rhabdoid histology in these *SMARCB1*-deficient NPLC-derived tumours. In accord with this, primary human AT/RT samples also exhibit an ESC-like gene expression DNA-methylation signature [320]. Thus, the AT/RT genome exhibits hypermethylated patterns resembling that of pluripotent stem cells. These stem-like DNA hypermethylation patterns affect the regulatory regions of multiple genes involved in neural differentiation. Hence, SMARCB1 loss impairs the removal of DNA methylation and blocks the regular progression of lineage commitment [319–321]. These findings were substantiated by further studies indicating that partial or complete SMARCB1 loss-of-function in human embryonic stem cell lines impairs neuronal differentiation [228, 229]. The blockage of neural differentiation driven by SMARCB1 loss-associated epigenetic dysregulation is essential for AT/RT tumorigenesis [328]. However, it is unclear if the mechanisms regulating differentiation blocks differ between AT/RT subgroups, as suggested by the differential subgroup-specific vulnerabilities to inhibitors and therapeutic drugs [26, 329–332].

#### Molecular subgroups of extracranial MRTs and cell-of-origin

RNA-sequencing indicated that two distinct subgroups of extracranial MRTs exist that are distinguishable by virtue of nearly 1000 differentially expressed genes [23]. In subgroup 1, the most significantly over-expressed genes were those encoding immunoglobulins and genes associated with BMP-signalling as well as differentiation. In subgroup 2, significantly overexpressed genes were linked to cell adhesion and migration, WNT signalling and cellular differentiation. In both subgroups, a significant proportion of overexpressed genes was linked to neural crest development and neural differentiation suggesting that the cells-of-origin of MRTs derive from the neural crest lineage [23]. MicroRNA profiles of the extracranial MRTs indicated pronounced similarities to those of pheochromocytomas and paragangliomas, which are also neural crest-derived tumours [23]. Furthermore, DNA methylation analysis of extracranial MRTs indicated two subgroups with distinct methylation profiles that correlated with age at diagnosis. Subgroup A exhibited higher overall promoter methylation at CpG-islands compared to the other subgroup B [23]. Correlating methylation subgroups to clinical patient data revealed an overrepresentation of patients older than one year in subgroup A [23]. Interestingly, the promoters of homeobox genes and tumour suppressor genes were disproportionately represented among those that acquired methylation in subgroup A [23].

Comparing the different subgroups of AT/RTs with extracranial MRTs revealed distinct similarities between MRTs and ATRT-MYCs including global DNA hypomethylation and overexpression of *HOX* genes and genes involved in mesenchymal development, distinguishing them from other AT/RT subgroups [304] (Sect. AT/RT subgroups).

In order to determine the origin of pediatric extracranial MRTs, Custers et al. [315] reconstructed the developmental relationship between MRT cells and normal tissues from the distribution of somatic mutations. These analyses indicated that pediatric extracranial MRT cells are phylogenetically related to the neural crest lineage, in particular to neural crest-derived Schwann cells, and that MRTs originate during fetal life [315]. Re-expression of *SMARCB1* in patient-derived MRT organoids consistently resulted in more differentiated cell types and promoted neural to mesenchymal conversion [315]. Thus, differentiation of neural crest cells and exit from pluripotency appear to be strongly dependent upon SMARCB1 activity [reviewed by 180].

### Mechanisms underlying *SMARCB1* activity as a tumour suppressor

Transcriptomic and epigenomic analyses of AT/RT samples have characterized the epigenetic alterations that take place following *SMARCB1* loss [24, 26, 284, 304]. The multitude of these changes implies a wide variety of mechanisms by which *SMARCB1* loss initiates cellular transformation and malignancy. A few of them have been identified as summarized in the following.

#### BAF and Polycomb complex antagonism

One of the mechanisms by which *SMARCB1* loss in MRT precursor cells leads to tumorigenesis is the disturbed balance between the BAF complex and the Polycomb repressive complex 2 (PRC2). This balance is however necessary to maintain chromatin topology [261, 284]. Indeed, *SMARCB1*-deficient MRT cells are unable to remove repressive PRC2-mediated histone modifications such as H3K27Me3 from tumour suppressor genes, for example *CDKN2A* (MIM #600160) [261]. The *CDKN2A* gene encodes p16 which is involved in the suppression of proliferation and acts as a cyclin-dependent kinase inhibitor that binds to CDK4/6 thereby impairing the activation of the CDK4/6-cyclin D1 complex [231, 333]. The active CDK4/6-cyclin D1 complex normally phosphorylates the retinoblastoma protein, which releases the transcription factor E2F1 to promote gene expression associated with S phase progression [334]. *SMARCB1*-deficient MRT cells exhibit considerably reduced p16 expression causing increased cellular proliferation due to unchecked S-phase progression [335, 336 reviewed by 20]. Upon re-expression of SMARCB1 in MRT cells, p16 expression increases due to restored BAF chromatin remodelling activity at regulatory genomic regions of p16 [233, 336]. Thus, SMARCB1 loss in MRT cells causes imbalance of the activity of BAF and PRC2 complexes, leading to an increase in repressive epigenetic marks by PRC2 [253, 261]. It is likely that, in addition to *CDKN2A*, other tumour suppressor genes are also repressed in *SMARCB1*-deficient cells in a similar manner. For example, the glioma pathogenesis-related protein 1 gene (*GLIPR1*; MIM #602692), which also acts as a tumour suppressor, shows strong promoter hypermethylation and is downregulated in AT/RT [24].

The balance between PRC2 and BAF activity is essential for tumour suppression by SMARCB1 [reviewed by 291, 337-339]. The Enhancer of zeste homolog 2 (*EZH2*; MIM #601573) encodes a histone-lysine N-methyltransferase and is an important component of PRC2. The inactivation of *Ezh2* in a conditional mouse model completely blocked tumour formation caused by *Smarcb1* inactivation [261]. In MRT cells, SMARCB1 loss leads to the increased expression and recruitment of EZH2 to PRC2 target genes which are H3K27-trimethylated and consequently in a repressed state [261, 284, 340–342]. In addition to *EZH2*, other genes encoding protein components of the PRC2 complex are overexpressed in AT/RT [24].

Remarkably, the active BAF complex is able to promote gene expression within a few minutes by removing PRC2 and its repressive H3K27me3 mark from promoters and enhancers [22]. Thus, the activity of the BAF complex opposes PRC2 on a minute-by-minute basis without any need for replication, polymerase occupancy or transcription in order to provide rapid epigenetic plasticity [reviewed by 20].

#### SMARCB1 inhibits the activation of *MYC* target genes

The SMARCB1 protein binds via its RPT1 domain to the C-terminus of the MYC protein, a master regulator of genome-wide transcription that potentiates oncogenic transformation when overexpressed [276, 343, 344]. The tumour-suppressor functions of SMARCB1 are mediated in part by inhibition of MYC binding to its target genes [320, reviewed by 345]. The analysis of MRT-derived organoids indicated that SMARCB1 loss during neural crest development prevents the inactivation of certain MYC enhancers, which is essential for proper lineage specification [346]. It has been suggested that SMARCB1 loss in MRTs leads to increased looping of these enhancers to the MYC promoter, thereby potentially activating its transcription [346]. Upon SMARCB1 reintroduction into MRT cells, MYC is displaced from chromatin genome-wide [347]. This activity of SMARCB1 is independent of its effects on chromatin remodelling within the BAF complex. Instead, SMARCB1 induces RNA polymerase pausing at genes regulated by MYC [348]. A key transcriptional function of MYC is to modulate release of paused RNA polymerases at MYC target genes and this activity is impaired by SMARCB1. Independent of any changes in MYC protein expression, the loss of SMARCB1 activates MYC at a functional level, leading to the activation of MYC target genes in MRT [348]. Thus, SMARCB1 antagonizes MYC and SMARCB1 loss drives malignancy via MYC overexpression.

#### SMARCB1 loss leads to WNT/beta-catenin hyperactivation

*Smarcb1* deficiency in the developing limb mesenchyme of conditional knock-out mice is responsible for the aberrant activation of the canonical Wingless-related integration site (WNT)-signalling pathway and leads to defects consistent with WNT/beta-catenin overexpression [349]. Re-expression of *SMARCB1* in *SMARCB1*-deficient MRT cell lines results in the down-regulation of beta-catenin target genes [349]. Transcriptome analysis of WNT pathway genes in AT/RT primary tissues and AT/RT cell lines indicated that the WNT family member 5B gene (*WNT5B*; MIM #606361) is significantly upregulated as compared with non-tumour brain samples [350]. The WNT5B protein binds to the protein product of the Frizzled class receptor 1 gene (*FZD1*; MIM #603408) and regulates the differential expression of downstream pathway genes [350]. WNT inhibitors decrease the proliferation of AT/RT cells suggesting that they might have future therapeutic potential [350].

In human embryonic stem cells (hPSCs), SMARCB1 loss-of-function leads to disturbed actin cytoskeleton organization, cell-cell interaction and cell-extracellular matrix (ECM) interaction associated with a significant reduction in beta-catenin levels. Thus, SMARCB1 is important for the regulation of cell-cell and cell-ECM interactions in hPSCs, at least in part mediated by the WNT signalling pathway [229].

#### SMARCB1 loss activates the hedgehog-GLI1 pathway

In a specific subgroup of AT/RTs, the SHH signal pathway is activated and hence SMARCB1 loss is associated with aberrant activation of this pathway [24, 351]. Hedgehog (HH) signalling has critical functions in cell proliferation and differentiation during development [reviewed by 307]. In mammals, there are three hedgehog genes: sonic hedgehog (*SHH*; MIM #600725), Indian hedgehog (*IHH*; MIM #600726) and desert hedgehog (*DHH*; MIM #605423). They are expressed in different tissues and at different stages of development, suggestive of different biological activities [reviewed by 352]. However, SHH is the most potent of these ligands and is widely expressed in adult tissues [reviewed by 307]. SHH signalling is crucial during embryonic development and for the maintenance of tissue polarity. Aberrant SHH signalling has been implicated in tumorigenesis for many different cancer types [reviewed by^307^, ^353^]. Hedgehog signal transduction is initiated by the binding of the HH ligands to the Patched-1 receptor encoded by the *PTCH1* gene (MIM #601309). The glioma-associated oncogene family zinc finger-1 (GLI1) is an important downstream effector in this signalling cascade. Importantly, SMARCB1 protein deficiency leads to aberrant activation of the HH-GLI1 pathway [351]. By using affinity purification–mass spectrometry and chromatin immunoprecipitation, SMARCB1 was found to localize upstream to the transcriptional start sites of the *GLI1* and *PTCH1* genes indicating very specific interactions [351]. Furthermore, small-hairpin-RNA-mediated knockdown of *Smarcb1* in mouse TM3 cells causes the upregulation of *Gli* and *Ptch1* expression, which leads to the activation of hedgehog signalling [351]. In accordance with this, re-expression of *SMARCB1* in MRT cells represses *GLI1* expression [351]. Therefore, the SMARCB1 protein acts as an important regulator of *GLI1* gene expression. In the subgroup of AT/RT with the molecular signature termed AT/RT-SHH, an enrichment of gene expression associated with SHH signalling pathway activation has been observed [24, 26]. The reason why *SMARCB1* loss does not activate the SHH pathway to the same extent in the other AT/RT subgroups remains unclear but is most likely related to different cellular origins for these subgroups [281] (Sect. Cell-of-origin of AT/RT subgroups).

### UPR activation and ER stress in *Smarcb1*-deficient cells

Several studies have suggested that BAF complexes are involved in the rewiring of cancer metabolism [reviewed by 354]. Epigenetic abnormalities deregulate metabolic enzymes or signalling pathways that are supportive of the survival and rapid proliferation of cancer cells. However, significant upregulation of protein anabolism can render cells susceptible to disruption of their proteostatic machinery. Importantly, *SMARCB1*-deficient MRT cells are highly sensitive to disturbances of protein homeostasis (proteostasis) as shown by treatment with proteasome inhibitors [355, reviewed by 330]. In order to investigate this in greater detail, Carugo et al. [356] generated embryonic murine mosaic models of liver MRT by introducing a tissue-specific Cre recombinase expressed from the murine albumin promoter via trans-uterine adenoviral injection in *Smarcb1*^*LoxP/LoxP*^ embryos at embryonic day E12.5. This was necessary in order to avoid early embryonic and perinatal lethality of classical or other conditional *Smarcb1* knockout mice which has impaired the analysis of the role of *Smarcb1* loss during tissue specification and mouse organogenesis [178, 357, 358]. Genetic mosaicism in their embryonic murine MRT model enabled Carugo et al. [356] to study the malignant properties of *Smarcb1*-deficient cells by bypassing the early lethality and allowing the tissue-specific time-restricted activation of a reporter gene and the quantification of tumour burden [356]. Specifically, the *in utero* mosaic Cre-mediated loss of *Smarcb1* targeted to E12.5 epithelial liver progenitor cells resulted in liver hyperplasia with severe dysplastic, degenerative changes and disruption of normal liver architecture [356]. The livers also had tumours that exhibited histopathological features of MRT with high proliferation activity. Transcriptome and protein expression analysis of these liver tumour cells indicated massive activation of the unfolded protein response (UPR) [356]. UPR becomes activated in response to the accumulation of unfolded or misfolded proteins in the lumen of the endoplasmic reticulum in order to restore normal cellular functions by preventing protein translation and the degradation of misfolded proteins. Upon UPR activation, signalling pathways are upregulated that lead to an increase of chaperones involved in protein folding. The activation of UPR is likely to be a by-product of the MYC-induced hypermetabolic state in the *Smarcb1*-deficient tumour cells [356]. In addition to massive UPR activation, *Smarcb1* loss also induced a robust ER stress response and autophagy [356]. Transcriptome analysis of Cre-induced *Smarcb*1-null liver tumours indicated significant upregulation of p53 pathways involved in the regulation of protein metabolism, ER stress adaptation and autophagy compared to controls. Based upon their findings, Carugo et al. [356] concluded that *Smarcb1* loss leads to the activation of different oncogenic pathways and induces UPR stress. Under these circumstances, p53 activation regulates cellular homeostasis, cell-cycle progression, cell survival, protein biosynthesis and removal by autophagy. Of note, rhabdoid tumours do not exhibit pathogenic p53 alterations [298]. Thus, p53 has a context-specific, pro-survival role in *Smarcb1*-deficient tumour cells and is an important regulator of proteostasis [356]. In accordance with this, interference with the cellular proteostatic machinery by drugs is highly lethal in the *Smarcb1*-deficient liver tumours and may represent a promising therapeutic target for MRTs [329, 356, reviewed by 330]. It should be noted that the survival of *SMARCB1*-deficient MRT cell lines is dependent upon DDB1-CUL4 Associated Factor 5 (DCAF5), a substrate receptor of the CUL4-DDB1 E3 ubiquitin-protein ligase complex, which targets specific proteins for ubiquitylation and degradation [reviewed by 359]. In the absence of SMARCB1, DCAF5 mediates the degradation of residual BAF complexes which is essential for the proliferation of SMARCB1-deficient MRT cells [359].

### Mutational profile in patients with *SMARCB1*-related SWN and RTPS1

#### Hypomorphic or semi-functional *SMARCB1* PVs in patients with SWN

The strikingly different tumour phenotypes in patients with *SMARCB1*-related SWN as compared to patients with RTPS1 indicate that the types and consequences of germline pathogenic *SMARCB1* variants must be different between these patient groups and will affect different tumour precursor cells. Additionally, the timing of the tumour-specific inactivation of the *SMARCB1* wild-type allele and the loss of additional tumour suppressor genes are responsible for the differences in tumorigenesis in SWN compared with RTPS1.

The pathogenic *SMARCB1* variants identified in patients with SWN are predominantly non-truncating mutations, including missense variants, in-frame deletions or splice‐site variants, with a tendency to accumulate in the 5′‐region or 3′‐end of the gene [28, 33–43]. By contrast, pathogenic *SMARCB1* variants in MRTs are located in all parts of the gene or delete all, or at least large parts of, the coding sequence [7, 10, 13, 40, 41, 297, 360, 361]. In most MRTs, the complete loss of nuclear SMARCB1 protein expression is a diagnostic marker [reviewed by 17]. According to the data available, there is a strong genotype/phenotype correlation in the sense that complete loss-of-function *SMARCB1* PVs are characteristic of MRT [24, 40, 41]. In contrast to this, most PVs in patients with *SMARCB1*-related SWN are likely to be semi-functional or may not affect all isoforms of *SMARCB1* leading to reduced SMARCB1 protein expression levels or only partial loss of SMARCB1 protein function [33–40]. The most common pathogenic *SMARCB1* variant identified so far in patients with SWN (c.*82C > T) is located within the 3’UTR [34, 38, 40, 42, 43, 362]. This 3’UTR variant leads to reduced *SMARCB1* expression levels due to lower mRNA stability [43, 363].

Even though most germline pathogenic *SMARCB1* variants causing SWN are non-truncating, germline *SMARCB1* PVs that generate a premature termination codon (PTC) have also been identified in patients with SWN. The majority of these are located in *SMARCB1* exon 1. PTCs located in *SMARCB1* exon 1 (c.30delC and c.34 C > T) of patients with classical SWN lead to transcripts that are not completely degraded but instead result in N-terminally truncated SMARCB1 proteins by translational reinitiation at a downstream AUG codon [364]. Furthermore, immunohistochemical analysis has indicated that N-terminally truncated SMARCB1 proteins are expressed in schwannomas of the respective patients harbouring exon 1 PVs [364]. The retention of partial activity of N-terminally truncated SMARCB1 has also been detected in a comprehensive deep mutational scanning study of *SMARCB1*, encompassing 8,418 amino acid substitutions, performed in order to assess their functional impact [273]. The residual activity of N-terminal nonsense PVs is due to alternative methionine start sites at residues 1–4, 27 and 38 in the N-terminus of SMARCB1, which enable downstream read-through [273]. This is in accordance with the reduced efficiency of nonsense-mediated decay (NMD) observed within 200 nucleotides of translational start codons [365].

This retention of partial function by N-terminal truncations mediated by the use of alternative methionine start sites may also explain the mosaic SMARCB1 protein expression pattern in schwannomas harbouring these variants. It is likely that these truncated SMARCB1 proteins are not fully stable thereby resulting in a mosaic SMARCB1 staining pattern. This has been observed and studied in detail in schwannomas of patients with the c.30delC and c.34 C > T pathogenic *SMARCB1* variants. Remarkably, pathogenic variants causing PTCs in *SMARCB1* exon 1 have not been reported in MRTs. These findings are in line with the concept that in contrast to the complete absence of SMARCB1 expression in MRT, altered SMARCB1 proteins with modified activity and reduced expression are responsible for a mosaic SMARCB1 expression pattern in the tumours of patients with schwannomatosis [364]. Indeed, several studies have observed that schwannomas in patients with germline *SMARCB1* PVs exhibit a mosaic SMARCB1 protein expression pattern as determined by immunohistochemistry [28, 363, 366, 367]. This mosaic pattern results from mixed immuno-positive and -negative nuclei, consistent with the expression of the SMARCB1 protein in a subset of tumour cells. This mosaic SMARCB1 expression is most likely related to the hypomorphic nature of the pathogenic variants in SWN patients that encode stable mRNA transcripts giving rise to detectable amounts of SMARCB1 protein. Since the wild-type *SMARCB1* allele is often lost in schwannomas of patients with schwannomatosis, the SMARCB1 protein detected in schwannoma cells must be encoded by the mutant allele. Our inability to detect mutant proteins in all tumour cells by immunostaining is most likely a consequence of the instability of mutant SMARCB1 proteins [364]. This instability results in immunologically non-reactive SMARCB1 protein degradation products in a proportion of the schwannoma cells. Since this degradation is probably a random process, some cells may still express detectable amounts of SMARCB1 protein resulting in a mosaic expression pattern when analysing schwannoma tissue sections. [144, 364, reviewed by 368].

#### *SMARCB1* missense variants in MRT

In contrast to schwannomas, complete loss of SMARCB1 protein expression as determined by immunohistochemistry is frequently observed in MRTs. In many instances, this is due to truncating PVs or loss of the complete *SMARCB1* gene leading to biallelic *SMARCB1* inactivation. However, missense PVs in specific *SMARCB1* domains have the potential to be similarly destructive [369]. This has been shown by deep mutational scanning of *SMARCB1* performed in order to assess the functional impact of 8,418 amino acid substitutions [273]. After prioritization, thirteen SMARCB1 amino acid residues intolerant to missense PVs were identified by expression of constructs containing these variants in *SMARCB1*-deficient tumour cell lines. Six of these missense PVs were located within the WHD domain (positions: R52, A55, I63, K77, L90 and L91), three within the IDR (positions: E122, Q123 and A125) and four within the RPT2 domain (positions: D277, W281, E300 and I315) [273]. Not unexpectedly, these missense PVs have not been observed in those patients with SWN that have been analysed to date. Of particular interest were the loss-of-function intolerant residues that are located in RPT2, which appear to facilitate important intramolecular interactions of SMARCB1 [273]. Remarkably, expression constructs containing the *SMARCB1* W281P and I315R missense variants exhibited functional properties similar to constructs with nonsense variants at the same positions as determined by reduced proliferation of MRT cell lines transfected with the respective expression constructs. In contrast to constructs with *SMARCB1* nonsense variants, the expression of the constructs with either the W281P or the I315R missense variants was readily detectable at both the RNA and protein level. Thus, these missense variants would appear to perturb the ability of SMARCB1 to enable BAF complex assembly and chromatin remodelling, without necessarily leading to complete protein degradation. Molecular dynamic modelling revealed that these missense mutants disrupt the flexibility of the N-terminal winged-helix domain of SMARCB1, suggesting a novel mechanism by which the SMARCB1 tumour suppressor function is disrupted. Indeed, these missense variants caused altered chromatin remodelling patterns, due to significant reduction in BAF complex activity, as well as changes in gene expression profiles in line with severely disturbed SMARCB1 protein function [273]. Thus, certain *SMARCB1* PVs result in loss-of-function even if they do not lead to complete loss of mutant SMARCB1 protein expression.

#### SMARCB1 functional loss in MRT due to nuclear export

In a subgroup of cranial MRT, the AT/RT-TYR subgroup, PVs leading to truncation or mutation of the C-terminal part of SMARCB1 are quite common (44%) [279]. Most of these PVs cause C-terminally truncated SMARCB1 proteins that are localized in the cytoplasm [279]. By contrast, wild-type SMARCB1 is a nuclear protein and loss of nuclear SMARCB1 staining is very frequent in AT/RT. Importantly, the SMARCB1 protein harbours a nuclear export signal (NES) within the RPT2 region [278] (Fig. 3). Remarkably, C-terminal truncation of SMARCB1 leads to the unmasking of the nuclear export sequence causing the cytoplasmic localization of SMARCB1 associated with the loss of tumour suppressor function [278, 279]. It has been estimated that 19% of all AT/RT exhibit cytoplasmic localization of SMARCB1 [279]. Whether nuclear export of mutant SMARCB1 also contributes to the development of schwannomas or other tumours in patients with *SMARCB1*-related SWN is currently unknown.

#### Non-coding *SMARCB1* PVs in patients with SWN

Pathogenic variants (PVs) in *LZTR1* or *SMARCB1* are detected in approximately 86% of familial and ∼40% of sporadic schwannomatosis cases utilizing standard clinical mutation analysis including exons and intronic segments at exon boundaries (typically ± 20 nucleotides) [33–36, 38, 40, 42, 65, 67, 68, 362, 363]. It has been argued that PVs in deep intronic or regulatory regions of both genes, regions that are not evaluated by exon-based sequencing strategies, might have been missed in the aforementioned studies and that this has inevitably resulted in a reduced pathogenic variant detection rate. In order to investigate whether PVs in deep intronic or regulatory regions of the *NF2*, *SMARCB1* and *LZTR1* genes contribute to the pathogenesis in patients with schwannomatosis, Piotrowski et al. [43] investigated 33 SWN patients without germline first-hit PVs in *NF2*, *SMARCB1* and *LZTR1* as determined by initial clinical exon sequencing. The analysis of the entire genomic region of these genes indicated deep intronic but clearly pathogenic *SMARCB1* variants in two of the 33 SWN patients (Supplementary Table 3). These authors identified five further intronic likely pathogenic variants in the three genes, one of them also in *SMARCB1*. Smith et al. [370] also identified a deep intronic *SMARCB1* PV (Supplementary Table 3) whilst Tauziède-Espariat et al. [371] identified a *SMARCB1* deep intronic pathogenic variant in intron 1 present in the germline of two unrelated young children with AT/RT and RTPS1. These findings indicate that deep intronic *SMARCB1* PVs can be disease-causing and that these regions should therefore be included in molecular diagnostic panels.

In addition to intronic PVs, those located in the 3’UTR are also an important cause of *SMARCB1*-related SWN indicating that this region should be included in the comprehensive screening of SWN patients in order to increase the pathogenic variant detection rate [34, 38, 40, 42, 43, 81, 362, 372].

#### Somatic mosaicism in *SMARCB1*-related SWN and RTPS1

Remarkably, the *SMARCB1* germline PV detection rate is much higher in familial SWN patients than in patients with sporadic *SMARCB1*-related SWN [32, 40]. Indeed, germline *SMARCB1* PVs account for up to 48% of familial SWN cases and 9.5% of sporadic SWN [33–36, 38, 40, 65, 67, 68, 363]. One possible explanation for this could be a high proportion of somatic mosaicism for *SMARCB1* PVs in sporadic patients, with variant allele frequencies being too low to be detected in blood. By contrast, the *SMARCB1* PV should be readily detectable in independent tumours of the patient harbouring somatic mosaicism for a *SMARCB1* PV. In order to address this, Smith et al. [81] analysed two independent schwannomas from 53 SWN patients who did not fulfil the diagnostic criteria for *NF2*-related SWN. Remarkably, 43% of patients with no identified germline *NF2* PVs in blood had low-level mosaicism for a pathogenic *NF2* gene variant and hence had mosaic *NF2*-related SWN. However, somatic or mosaic *SMARCB1* or *LZTR1* PVs were not identified in the remaining patients without germline PVs in one of their SWN genes. These findings suggest that somatic mosaicism for *SMARCB1* (and *LZTR1*) is uncommon in SWN patients.

By contrast, somatic *SMARCB1* PVs causing mosaicism are more frequent than previously assumed in children with rhabdoid tumours [183]. It is estimated that 6–21% of these children exhibit somatic mosaicism for a *SMARCB1* PV as determined by sequence analysis with high sensitivity to detect low-level mosaicism [373, 374]. Gonadal mosaicism of the parents of patients with rhabdoid tumours is also not uncommon and has been observed in several studies [5, 6, 12, 13, 53, 375]. A case of maternal germ line mosaicism has also been reported in *SMARCB1-*related SWN [376].

### Mouse models of *Smarcb1* loss leading to either schwannomas or rhabdoid tumours

#### The time window of *SMARCB1* inactivation in rhabdoid tumour development

Both the type of pathogenic *SMARCB1* variant and whether it leads to the complete loss or only the partial loss-of-function determines which type of tumour develops, either rhabdoid tumour (RT) or schwannoma. The time window of complete *SMARCB1* inactivation during development is also important in this context. This has been ascertained through the analysis of conditional *Smarcb1* knockout-mouse models [178, 179]. Homozygous germline inactivation of *Smarcb1* in mice is embryonic lethal; nullizygous animals (*Smarcb1*^−/−^) are not born after heterozygous inter-crosses. *Smarcb1*^−/−^ embryos develop to the blastocyst stage but die shortly after implantation before E6.5 [357, 377, 378]. Heterozygous *Smarcb1*^*+/−*^ mice are born and appear to be normal but, starting at the age of a few weeks, they develop extracranial sarcomas resembling human RTs mainly in soft tissues derived from the first branchial arch with a long latency and weak penetrance [357]. Only tissue- and developmental stage-specific conditional *Smarcb1* knockout mouse models using different promoters to induce Cre-mediated *Smarcb1* deletion succeeded in generating a murine model for AT/RT [178, 179, reviewed by 180]. By means of temporal control of tamoxifen injection in *Smarcb1*^flox/flox^;Rosa26-Cre^ERT2^ mice, the phenotypes associated with *Smarcb1* inactivation at different developmental stages could be investigated [178]. Injection before E6, at birth or when the mice were two months of age, caused lethality, hepatic toxicity or development of T-cell lymphomas [178]. However, tamoxifen injection and thus biallelic *Smarcb1* loss between E6 and E10 resulted in viable mice which developed mainly intracranial tumours with high penetrance and rapid onset. These tumours exhibited anatomical, morphological and gene expression profiles comparable to those of human AT/RTs [24, 178]. By using the protein zero (P0)-promoter to activate the Cre recombinase and thus *Smarcb1* loss at E9.5 in neural crest cells, Vitte et al. [179] also succeeded in generating a conditional knockout mouse model of cranial rhabdoid tumours that resembles human AT/RTs. These *P0-CreC; Smarcb1*^flox/flox^ mice were viable and developed RTs in cranial nerves and meninges. The tumours displayed typical histological and immunohistochemical features of human RTs [179]. Expression profiling indicated that tumours of the *P0-CreC*;*Smarcb1*^flox/flox^ mice recapitulate the molecular diversity of human AT/RTs [179]. Importantly, both human AT/RTs and the rhabdoid tumours from the *P0-CreC; Smarcb1*^flox/flox^ mice exhibit specific gene expression markers of early neural crest formation indicative of an early neural crest cell as the cell-of-origin [179].

Taken together, the development of AT/RT is time-dependent in the sense that a specific developmental time window exists during which the tumour progenitor cell is vulnerable to complete *Smarcb1* loss initiating rhabdoid tumour growth. It is highly likely that during human embryonic development, *SMARCB1* loss in neural crest cells or neural progenitor cells during a specific window of time associated with the disturbance of neural differentiation is a critical step in the tumorigenesis of AT/RTs [178, 179, reviewed by 180] (see Sect. Blocked neural differentiation in AT/RTs). The existence of an early narrow spatio-temporal window during which complete *Smarcb1* loss results in malignant transformation may explain why some *SMARCB1* mutation carriers in RTPS1 families do not develop rhabdoid tumours (Sect. Co-occurrence of RTPS1 and schwannomatosis in families). If a sensitive time period during the early development of neural crest cells is completed without biallelic complete *SMARCB1* inactivation, AT/RT tumorigenesis is not initiated. In other words, the absence of AT/RT in carriers of loss-of function germline *SMARCB1* PV is explicable in terms of the retention of the *SMARCB1* wild-type allele and its activity in neural crest cells or neural progenitor cells during a vulnerable early stage of embryonic development.

#### Mouse model of *SMARCB1*-related schwannomas

In schwannomas of patients with schwannomatosis, somatic biallelic *NF2* gene inactivation is very frequent [34, 35, 65]. Indeed, loss-of-function of *NF2* appears to be important for schwannoma growth. This conclusion is in accord with the finding that conditional *Smarcb1* knockout mice do not develop schwannomas, indicating that biallelic *Smarcb1* loss is not on its own sufficient for the growth of schwannomas [178, 179]. In order to investigate the co-involvement of *Smarcb1* and *Nf2* in the pathogenesis underlying schwannomas, Vitte et al. [179] generated conditional knockout mouse models of *Smarcb1* and concomitant *Nf2* gene loss. First, they created *P0-CreC; Smarcb1*^flox/flox^;*Nf2*^flox/flox^ mice which were not viable, indicating that the loss of both *Smarcb1* and *Nf2* during early development (E9.5) is lethal. However, by using a different CRE-promoter (mGFAP) which is expressed later in embryonic development than the P0-promoter, mice with the combination of *Smarcb1* and *Nf2* inactivation became viable. In these *mGFAP-Cre; Smarcb1*^flox/flox^;*Nf2*^flox/flox^ mice, Cre is expressed in the dorsal root ganglia starting at E13.5. The *mGFAP-Cre; Smarcb1*^flox/flox^;*Nf2*^flox/flox^ mice developed tumorlets consisting of Schwann cells in the dorsal root ganglia, reminiscent of the schwannoma tumorlets found in *NF2* patients [179]. These tumorlets are considered to be small schwannomas exhibiting biallelic *NF2* inactivation and occur mainly in the spinal nerve roots [379]. Importantly, schwannoma tumorlets were not found in the dorsal root ganglia of *mGFAP-Cre; Smarcb1*^flox/flox^;*Nf2*^flox/+^ mice, indicating that biallelic *NF2* loss is essential for schwannoma formation [179]. Similar to the situation in schwannomatosis patients, the loss of both *Smarcb1* and *Nf2* did not increase the malignancy of the tumours in *mGFAP-Cre; Smarcb1*^flox/flox^;*Nf2*^flox/flox^ mice as compared to tumours in *mGFAP-Cre; Nf2* ^flox/flox^ mice with biallelic *Nf2* loss but retention of *Smarcb1* activity.

Taken together, the conditional knockout mouse models established by Vitte et al. [179] indicated that *Smarcb1* loss at a later developmental stage (starting at E13.5) in the Schwann cell lineage, in addition to biallelic *Nf2* gene inactivation, results in schwannomas in *mGFAP-Cre; Smarcb1*^flox/flox^;*Nf2*^flox/flox^ mice. This mouse model of schwannoma development impressively reproduces the genetic profile of schwannomatosis-associated schwannomas with concomitant loss of both *Smarcb1* and *Nf2* [179]. *Smarcb1* loss and biallelic *Nf2* inactivation at later stages of Schwann cell development (starting at E13.5) lead to benign schwannomas but not rhabdoid tumours. By contrast, conditional biallelic *Smarcb1* knockout during a narrow time window of early neural crest cell development (E6-E10) results in rhabdoid tumour growth [178, 179]. Thus, Schwann cell differentiation suppresses *Smarcb1-*driven malignant tumorigenesis in this mouse model.

### Co-involvement of several tumour suppressor genes in schwannoma development

**Fig. 4:**
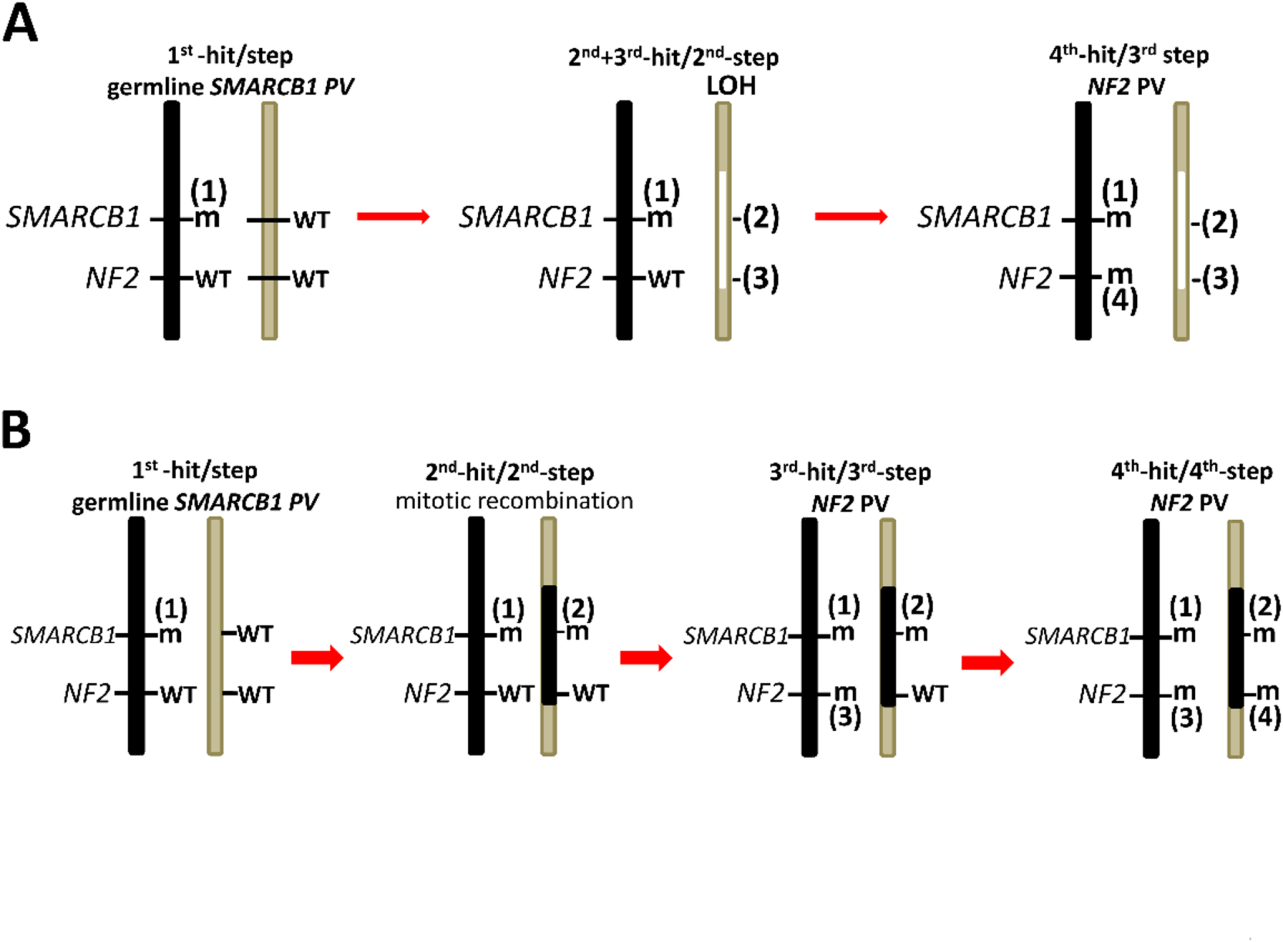
**A** Four-hit/three-step model of tumorigenesis in patients with a germline *SMARCB1* pathogenic variant (PV) (first hit and step). The second step involves loss of heterozygosity (LOH) of 22q which serves to remove the wild-type *SMARCB1* allele and one of the two *NF2* alleles located in *trans* to the germline *SMARCB1* PV. The third step is the somatic mutation of the other *NF2* allele located on the chromosome harbouring the germline *SMARCB1* mutation. **B** If mitotic recombination were to represent the second step, this would lead to a reduplication of the chromosomal region with the germline *SMARCB1* PV allele. However, the *NF2* gene would not be deleted by this event. Instead, two wild-type *NF2* alleles would still be present. The biallelic inactivation of *NF2* would require two independent *NF2* PVs (third and fourth mutational steps). Thus, mitotic recombination is not compatible with the four-hit/three-step model of tumorigenesis. Instead, a four-hit/four-step model of tumorigenesis would have to be postulated. m: mutant allele; WT: wild-type allele

The molecular mechanism underlying the tumorigenesis of schwannomas in patients with *SMARCB1*- (and *LZTR1-*) related SWN is clearly not in agreement with the classic Knudson two‐hit model hypothesis involving the biallelic inactivation of a single tumour suppressor gene. Instead, tumorigenesis of schwannomas in patients with *SMARCB1*‐ (and *LZTR1-*) related SWN appears to follow a four‐hit/three‐step model that includes somatic biallelic inactivation of the *NF2* gene [35, 368]. In the following, this model will be explained with a focus on *SMARCB1*-related SWN although it may also be applied in an analogous manner for *LZTR1*-related SWN. The basis of the four-hit/three-step model is that all three tumour suppressor genes known to be relevant to schwannoma development, namely *NF2*, *LZTR1* and *SMARCB1*, are located on chromosome 22q (Fig. 2). Furthermore, chromosome 22q loss-of-heterozygosity (LOH) is a frequent somatic event in schwannomas [33–35, 42, 65, 81, 380]. In patients with *SMARCB1*-related SWN, the germline *SMARCB1* PV is considered to be the **first hit (first step)**. The **second and third hit (second step**) then involves loss-of-heterozygosity (LOH) of 22q associated with the loss of the wild-type *SMARCB1* allele and one of the two *NF2* alleles (Fig. 4A). The tumour-specific 22q LOH is caused by deletions of different sizes on the long arm of chromosome 22 (22q) [28, 33–35, 42, 65, 380]. Importantly, the 22q LOH affects the chromosome harbouring the wild-type *SMARCB1*-allele and hence occurs in *trans* to the germline *SMARCB1* PV which is retained in the schwannoma. Finally, the **fourth hit (third step**) involves a tumour-specific pathogenic variant of the *NF2* gene located in *cis* to the *SMARCB1* germline PV that leads to biallelic *NF2* inactivation driving schwannoma development (Fig. 4A). Schwannomas in patients with schwannomatosis frequently exhibit tumour-specific intragenic *NF2* PVs that are different in anatomically distinct schwannomas of a given patient, indicative of their somatic origin [33–35, 62, 381]. The chronological order of the tumour-specific alterations, namely 22q LOH and somatic *NF2* PVs, is probably not fixed and may be interchangeable. Moreover, these different genetic events may influence each other in the sense that biallelic loss of *SMARCB1* and a dosage loss (haploinsufficiency) of half of the *NF2* gene due to 22q LOH may cause Schwann cell proliferation that then accelerates mutagenesis giving rise to the somatic intragenic *NF2* PV [380]. In any case, the key point to be taken from the four-hit/three-step model is that biallelic *SMARCB1* loss is insufficient for schwannoma growth in patients with *SMARCB1*-related SWN. Additional inactivation of the *NF2* gene is also necessary for schwannoma development. The co-involvement of *SMARCB1* and *NF2* in the pathogenesis underlying schwannomas in patients with *SMARCB1*-related SWN is substantiated by mouse models of schwannoma development [179] (Sect. Mouse model of *SMARCB1*-related schwannomas). The fact that *NF2* loss is important for the development of schwannomas is also reflected in the observation that at least 50–75% of sporadic schwannomas exhibit somatic pathogenic *NF2* variants [380, 382, 383].

In patients with *SMARCB1*-related SWN, the four-hit/three‐step model appears to underlie the vast majority of schwannomas (Fig. 4A). In the study of Piotrowski et al. [65], all 17 schwannomas from 9 patients with *SMARCB1*-related SWN exhibited somatic chromosome 22q LOH leading to *SMARCB1* and *NF2* loss. Further, all 17 schwannomas also exhibited somatic intragenic *NF2* PVs. It is likely that the four-hit/three-step model of tumorigenesis also accounts for other tumours in patients with *SMARCB1*-related SWN including meningioma and leiomyoma [55, 138].

Several studies have indicated that the proportion of schwannomas in patients with *SMARCB1*-related SWN exhibiting chromosome 22q LOH (chr.22q-LOH) is very high [34, 35, 65]. Of note, mitotic recombination has been excluded as the mechanism causing 22q LOH in schwannomas of patients with germline *SMARCB1* PVs [380]. Mitotic recombination as a causative mechanism would in any case not be compatible with the four-hit/three-step model of tumorigenesis because a reduplication of the chromosomal region with the germline *SMARCB1* PV allele by mitotic recombination would require two independent *NF2* PVs in both alleles for complete inactivation of *NF2* (Fig. 4B). Consequently, a four-hit/four-step model of tumorigenesis would have to be postulated which is possible but less likely than the four-hit/three-step model (Fig. 4B). More likely, as suggested by Hadfield et al. [380], a three-step model of schwannoma development, comprising the biallelic loss of *SMARCB1* and loss of one *NF2* allele mediated by 22q deletion, would serve to accelerate Schwann cell proliferation thereby driving the somatic intragenic *NF2* PV that represents the third step.

Instead of mitotic recombination, the frequent chromosome 22q LOH in schwannomas is most likely due to differentially sized deletions of parts of 22q. The mechanism underlying these deletions is unknown. It is however unlikely that non-allelic homologous recombination (NAHR), mediated by the multiple duplicated sequences on chromosome 22, the low copy repeats 22 (LCR22), is responsible for the deletions because the deletion breakpoints do not coincide with the positions of the LCR22 repeats which are located within a 6.5-Mb region on 22q11.2 [reviewed by 220] (Fig. 2). The majority of the somatic deletions of chromosome 22q observed in schwannomas of patients with *SMARCB1*-related SWN extend beyond this 6.5 Mb region [43, 65, 380]. Further, many of the somatic 22q deletions in schwannomas exhibit heterogeneous breakpoints located outside of the LCR22 sequences suggesting that these LCRs are not directly involved in deletion formation.

In addition to *SMARCB1* and *LZTR1*, other schwannomatosis predisposition genes located on chromosome 22q are likely to exist (Supplementary Table 2). In a proportion of schwannomatosis patients without identifiable germline *LZTR1* or *SMARCB1* PV, somatic 22q LOH has been detected in schwannomas with or without an identifiable somatic *NF2* PV [43]. Patients with this mutational pattern exhibit 22q-related SWN [66] (Table 1). The targeted sequencing of specifically chromosome 22q in 31 patients with 22q-related SWN has indicated five genes on chromosome 22 that might qualify as additional schwannomatosis predisposition genes (Supplementary Table 2), but further verification is required by the analysis of additional patients [43].

### Molecular signature of SWN-schwannomas

Biallelic loss-of function of the *NF2* gene is the initiating tumorigenic event of schwannomas in patients with *NF2*-related SWN and also in a large proportion of sporadic schwannomas [reviewed by 384]. Likewise, schwannomas of patients with *SMARCB1*- and *LZTR1*-related SWN exhibit biallelic *NF2* gene inactivation as mentioned in the previous section. Thus, loss of *NF2* gene function is a crucial event initiating tumorigenesis in most schwannomas. Furthermore, schwannomas of patients with *SMARCB1*- and *LZTR1*-related SWN are phenotypically and histopathologically indistinguishable from schwannomas of patients with *NF2*-related SWN and sporadic schwannomas. Nevertheless, it is plausible that the events which drive schwannoma growth differ depending upon the presence or absence of a germline PV in one of the three schwannoma predisposition genes (*NF2*,* SMARCB1* and *LZTR1*). In view of the genetic heterogeneity, it might be expected that the molecular signature including changes in DNA methylation and gene expression associated with alterations in signalling pathways varies comparing sporadic schwannomas and those of patients with either *NF2*-, *LZTR1*- or *SMARCB*1-related SWN. However, the vast majority of previous studies that addressed the genomic, gene expression and DNA methylation profiles of schwannomas have been focussed on sporadic schwannomas or those of patients with *NF2*-related SWN [384-387]. By contrast, the molecular signature of schwannomas from patients with genetically confirmed *LZTR1*- or *SMARCB1*-related SWN has been analysed so far only in a single study [111]. Perhaps suprisingly, DNA methylation profiling did not indicate clear differences between schwannomatosis-associated schwannomas (SWN-schwannomas) and sporadic schwannomas [111]. Furthermore, no significant differences in the DNA methylation profiles of SWN-schwannomas were detected when comparing tumours from patients with germline PVs in either *LZTR1* or *SMARCB1* [111]. However, four different DNA methylation subgroups were identified in SWN-associated schwannomas, which were specifically associated with the anatomic location of the tumours. Multiple schwannomas resected from different anatomic areas of the same patient resolved into different methylation clusters. This finding suggests that Schwann cells derived from different regions of the body exhibit different DNA methylation profiles [111]. Moreover, each methylation cluster exhibited a distinct transcriptome profile with upregulated expression of specific pathways including cAMP, NFkB, RB and PIGF. These findings indicate the putative existence of four subtypes of SWN-schwannomas [111]. Further, pathway analysis indicated the upregulation of VEGF, SHH and MEK pathways, in addition to mismatch-repair and DNA repair-related genes in SWN-schwannomas as compared to sporadic schwannomas [111]. Remarkably, SWN-schwannomas exhibited a significantly elevated number of chromosomal copy number variants and higher rates of chromosome 22q loss-of-heterozygosity as compared to sporadic schwannomas [111]. Taken together, these findings suggest that substantial differences may exist in the pathogenesis of sporadic versus SWN-schwannomas. However, significant differences in the molecular profiles of schwannomas derived from patients with either *LZTR1*- or *SMARCB*1-related SWN were not obvious. Mansouri et al. [111] investigated 25 schwannomas from 10 patients with *SMARCB*1-related SWN and 69 schwannomas from 26 patients with *LZTR1*-related SWN. Most likely, larger numbers of SWN-schwannomas should be analysed comparatively in order to identify any molecular differences between schwannomas derived from patients with either *LZTR1*- or *SMARCB1*-related SWN.

Single-cell RNA sequencing (scRNA-seq) of 22 schwannomas including sporadic tumours and those from patients with *NF2-*related SWN and non-*NF2*-related SWN indicated intra- and inter-tumoral transcriptional heterogeneity of schwannomas [388]. Nevertheless, six recurring distinct transcriptional programs (meta-programs) have been identified with gene signatures related to stress, myelin production, antigen presentation, interferon signalling, glycolysis and extracellular matrix [388]. The advantage of scRNA-seq is that intra-tumoral heterogeneity (ITH), which is a property of many tumours driven by genetic, epigenetic and microenvironmental influences, can be assessed very efficiently. Numerous scRNA-seq analyses of different tumour types indicated that ITH is often associated with “expression programs” comprising dozens of genes with coordinated variability in their expression across malignant cells within a given tumour. Similar ITH programs have been identified across tumours of the same cancer type, and in some instances even across different tumour types suggesting that ITH expression programs reflect basic principles of tumour biology. The consensus among related ITH programs from different tumours has been designated as meta-programs [389]. In schwannomas, six distinct gene expression meta-programs were identified [388]. Remarkably, these meta-programs were observed in schwannomas of different genetic backgrounds and from different anatomical locations. However, a clear clustering of schwannomas according to their genetic background and anatomical location was not possible suggesting that the schwannomas exhibit similar overall expression profiles. Unfortunately, no distinction was made between *SMARCB1*- or *LZTR1*-related SWN in this study [388]. Additional scRNA-seq analyses of schwannomas from patients with characterized germline variants in either *SMARCB1* or *LZTR1* would be instructive in order to elaborate any transcriptome differences that remain to be identified.

#### Recurrent *SH3PXD2A-HTRA1* fusion in SWN-schwannomas

Remarkably, a recurrent somatic fusion gene has been identified in 10% of sporadic schwannomas and in schwannomas of patients with *NF2*-related SWN [390]. The in-frame fusion involves the SH3 and PX domains-containing protein 2 A gene (*SH3PXD2A*; MIM #619455) and the high temperature requirement A serine peptidase 1 gene (*HTRA1*; MIM #602194), both located on chromosome 10q. The fusion results from a balanced 19-Mb inversion within chromosome 10q [390]. In vitro transfection assays indicated that the overexpression of the SH3PXD2A-HTRA1 fusion protein caused increased levels of phosphorylated extracellular signal-regulated kinase (ERK) indicative of an activated mitogen-activated protein kinase (MAPK) pathway. Further, the *SH3PXD2A-HTRA1* fusion has been shown to increase proliferation and invasive growth [390]. The *SH3PXD2A-HTRA1* fusion gene is also present in a subset of schwannomas from patients with schwannomatosis [111]. The *SH3PXD2A-HTRA1* fusion gene was detected in 2/24 (8.3%) of schwannomas from patients with *SMARCB1*-related SWN, and in 13/64 (20.3%) of schwannomas from patients with *LZTR1*-related SWN which is not significantly different [111]. In schwannomas of patients with *LZTR1*-related SWN, the fusion gene was significantly more frequent in painful schwannomas as compared to pain-free schwannomas. Furthermore, upregulation of the RAS/MAPK pathway was observed in schwannomas of patients with *LZTR1*-related SWN [111]. The RAS/MAPK pathway is also activated in sporadic schwannomas [390]. However, it is unknown if RAS/MAPK pathway activation also plays an important role in schwannomas of patients with *SMARCB1*-related SWN. Further studies, including higher numbers of schwannomas from patients with *SMARCB1*-related SWN, will be necessary to identify the full spectrum of pathways altered in these tumours.

### Molecular pathogenesis of neurodevelopmental disorders caused by germline *SMARCB1* PVs

In addition to its role as a tumour suppressor, the SMARCB1 protein is also an important regulator during development. *Smarcb1* is required for early embryonic survival since homozygous *Smarcb1*-null mouse embryos die between embryonic days 3.5 and 5.5 *post coitum* [357, 377, 378]. Human induced pluripotent stem cells and organoid models indicated that SMARCB1 loss during neuronal differentiation leads to a lack of stability among neural progenitor cells and a failure in maturation [319, 320]. Furthermore, significant differences in the response of cells to SMARCB1 loss were detected at different stages of neural differentiation, indicating a narrow time window early in neural commitment during which cells are highly vulnerable to SMARCB1 loss-of-function, exhibiting severe defects in the progression of differentiation [319, 320]. This is in accord with the results of inducible *Smarcb1* knockout mouse models [178] (Sect. The time window of *SMARCB1* inactivation in rhabdoid tumour development). In human embryonic stem cells, SMARCB1 is required for increased accessibility of chromatin regions associated with neural differentiation but dispensable for mesodermal or endodermal differentiation [228, 229]. In similar vein, induced loss of Smarcb1 protein in mouse embryonic stem cells impaired the expression regulation of genes associated with nervous system development [327]. An important role of SMARCB1 during human neurodevelopment may also be deduced from the observation that germline pathogenic *SMARCB1* variants cause some of the SWI/SNF-related intellectual disability disorders (SSRIDDs), which result from dysfunction of BAF complexes [reviewed by 211, 44, 216, 391] as reviewed in the following section.

#### Molecular pathogenesis of Coffin-Siris syndrome (CSS)

So far, 14 different intragenic *SMARCB1* PVs affecting single nucleotides and one partial gene deletion of 9-kb encompassing *SMARCB1* exons 8 and 9 as well as the 3’ flanking region of *SMARCB1*, have been identified in 35 patients with CSS [45, 46, 48, 49, 202–209, 282, 392, 394] (Table 4). The most common recurrent CSS-associated *SMARCB1* PV is an in-frame deletion of a single lysine, K364del (identified in 14 unrelated CSS patients). This recurrent PV is located in the C-terminal coiled-coil domain (CTD) of *SMARCB1* close to other missense PVs (Table 4). The CSS-causing *SMARCB1* variants cluster closely together at exons 8 and 9, indicating a specific position effect in the pathogenesis of *SMARCB1*-related CSS. It should be noted that the PVs in the *SMARCB1* CTD are not only found in the germline of patients with CSS but are also observed as somatic variants in different types of cancer [134, 283].

It is intriguing that PVs affecting single amino acids located within the CTD of *SMARCB1* cause a severe neurodevelopmental disorder such as CSS, but are not associated with rhabdoid tumour development. This is suggestive of a very specific role for the *SMARCB1* CTD which encompasses an alpha helical domain within a region of densely packed basic and positively-charged amino acids. Importantly, this alpha-helical domain directly binds to the acidic patch of the nucleosome [258]. CSS-causing PVs located within the *SMARCB1* CTD do not grossly alter the secondary structure of this domain. Instead, they disrupt nucleosome binding and preclude BAF-mediated nucleosome remodelling and DNA accessibility at enhancer regions [258]. The genome-wide localization of the BAF complex is not affected by PVs in the C-terminal alpha-helical domain of *SMARCB1*. Nevertheless, these complexes are defective in activating critical target genes [258, 259].

Among the CSS-causing PVs located in the *SMARCB1* CTD is the missense variant R377H [45, 394]. Re-expression of this variant in the *SMARCB1*-deficient G401 cell line did not result in any decrease in cell proliferation, in contrast to the nonsense R377* mutant which caused a significant reduction in cell proliferation [273]. These findings imply that the nonsense R377* mutation partially compromises *SMARCB1* tumour suppressor function, whereas the R377H missense PV retains this functionality [273]. This finding may explain why patients with CSS are not affected by pediatric AT/RT or other malignant tumours associated with complete loss of *SMARCB1* function.

Importantly, heterozygous CSS-causing *SMARCB1* PVs located in the CTD result in gene regulatory and morphological changes during induced pluripotent stem cell (iPSC)-neuronal differentiation. Indeed, differentiated neurons derived from iPSCs harbouring the heterozygous *SMARCB1* c.1091_1093del (p.K364del) variant showed less neurite outgrowth than wild-type controls [258, 259].

The conclusion that neurite outgrowth deficits may result from C-terminal non-truncating pathogenic *SMARCB1* variants has been confirmed by means of the genetically engineered mouse model created by Brugmans et al. [395]. These mice harboured a deletion of a cytosine in exon 9 at position c.1148 of *Smarcb1*, causing a frameshift of 36 amino acids until a downstream stop codon (c.1148del; p.P383QfsX36) [395]. Adolescent *Smarcb1*^1148del/1148del^ mice exhibited delayed weight gain and hydrocephalus including enlarged lateral ventricles. In their embryonic and neonatal stages, the brains of these mutant mice did not differ anatomically or histologically from the brains of wild-type controls [395]. Transcriptome analysis by single-cell RNA sequencing of brains from newborn mutant mice indicated that a complete brain is formed with all cell types from a normal mouse brain. Nevertheless, neuronal signalling was perturbed in these newborn mutant mice. Drastically lowered expression of the AP-1 transcription factor family was noted to be the cause of reduced expression of essential regulators of neurite outgrowth via growth cones in the mutant mice [395]. These findings are indicative of the important role of *SMARCB1* during neurodevelopment. Impaired *SMARCB1* function may also disturb neurite outgrowth and synapse formation in humans causing intellectual disability in patients with neurodevelopmental disorders such as CSS. This is in line with other studies showing neurite outgrowth deficits in neurons with pathogenic variants in other BAF complex genes [269, 396]. Taken together, deficiencies of BAF complex subunits play an important role in the pathogenesis of a subgroup of neurodevelopmental disorders. Indeed, BAF complex genes are the most frequently mutated genes among those involved in chromatin regulation in the context of neurodevelopmental disorders [259].

Importantly, BAF complex subunits are expressed in a temporal and cell-type specific manner during neurodevelopment. The BAF complex begins to switch subunits to those unique to neural progenitors, followed by subunits specific to neurons during differentiation from embryonic stem cells into neurons [263, 270]. Thus, the timely expression of these BAF subunits is essential for regulating cell fate during neurodevelopment. The combinatorial assembly of subunits determines cell lineage specification by creating specific patterns of chromatin states at different developmental stages, which are essential for normal neurodevelopment [reviewed by 397]. The pathogenesis underlying the SWI/SNF-related intellectual disability disorders (SSRIDDs) including CSS indicates that dysfunction of any of these subunits disturbs neural development and results in the overlapping clinical phenotypes of SSIDRs [reviewed by 211, 216, 391]. Since the clinical phenotype of SSIDRs is in several instances overlapping, genetic testing has become necessary in order to arrive at a correct differential diagnosis. For example, patients initially clinically diagnosed with Aicardi syndrome or Nicolaides–Baraitser syndrome have been reclassified as CSS cases after the identification of *SMARCB1* PVs in C-terminal domain which have been previously identified as recurrent mutations in patients with classical CSS [49, 393].

The important role of *SMARCB1* during nervous system development also became obvious through the conditional knockout mouse models generated by Vitte et al. [179]. In these mice, *Smarcb1* deletion was induced by the Cre promoters DHH and mGFAP later during Schwann cell development (beginning at E12.5 or E13.5, respectively). These *DHH-CreC; Smarcb* ^*flox/flox*^, *mGFAP-Cre; Smarcb1*^*flox/flo*x^ and *mGFAP-Cre; Smarcb1*^*del/flox*^ mice survived only a few weeks and developed progressive hindlimb paralysis. Their sciatic nerves were thinner and more transparent than those in control mice. Histological analysis of the nerve fibres indicated severe disturbances of structure and organization caused by *Smarcb1* loss [179]. These mice did not develop tumours indicating that *Smarcb1* loss during later stages of development is on its own not tumorigenic (see Sect. The time window of *SMARCB1* inactivation in rhabdoid tumour development). The severe neurological phenotype of these mice implies that SMARCB1 protein deficiency during early development of the human nervous system may well be responsible for the neurological deficits observed in patients with pathogenic *SMARCB1* variants and CSS [213, 214].

#### Patients with CSS and schwannomatosis

Remarkably, two patients have been identified who had both CSS and schwannomatosis [282, 392]. The 28 year-old female patient reported by Gallagher et al. [392] had multiple intra-thoracic schwannomas and a large painful schwannoma of the left upper arm as well as a severe clinical manifestation of CSS. Sequence analysis indicated a *de novo* germline in-frame deletion in *SMARCB1*, c.1091_1093del (p.K364del), which represents the most common recurrent *SMARCB1* PV in patients with CSS (Table 4). As mentioned in Sect. Molecular pathogenesis of Coffin-Siris syndrome (CSS), this pathogenic *SMARCB1* variant is located in the C-terminal domain. It has been shown that this PV impairs the nucleosomal binding of *SMARCB1* and leads to changes in gene expression as well as cellular morphology during induced IPSC differentiation which showed less neurite outgrowth than wild-type controls [258, 259].

The patient reported by Gossai et al. [282] harboured the recurrent pathogenic *SMARCB1* variant c.1121G > A (p.Arg374Gln) which also prevents the nucleosomal binding of *SMARCB1* [258, 259]. The analysis of a schwannoma in this patient indicated somatic loss of the wild-type *SMARCB1* allele, in combination with *NF2* loss [282]. The patient had a very severe form of CSS and also schwannomatosis with multiple spinal schwannomas and bilateral cranial nerve involvement [282]. Only 8% of patients with schwannomatosis exhibit single non-vestibular cranial schwannomas [29]. The severe phenotype of both patients suggests that at least some patients with CSS and *SMARCB1* PVs are at risk of developing schwannomas and should be investigated by MRI to prevent severe problems caused by a delayed diagnosis of the tumours.

#### Molecular pathogenesis of intellectual disability with choroid plexus hyperplasia (ID-CPH)

The pathogenic *de novo* missense *SMARCB1* variant (c.110G > A; p.Arg37His) identified in four patients with severe intellectual disability, choroid plexus hyperplasia and resultant hydrocephalus termed ID-CPH is located within exon 2 of *SMARCB1*, encoding the winged-helix DNA-binding domain (WHD) [217]. It remains to be determined how pathogenic variants in different parts of *SMARCB1* can lead to clinically different disorders of neurological development associated with severe intellectual deficits such as CSS and ID-CPH. The R37H missense variant located within the N-terminal *SMARCB1* WHD in patients with ID-CPH does not impair the ability of the SMARCB1 protein to bind to nucleosomes, as it has been shown for PVs located in the C-terminal domain of *SMARCB1* in patients with CSS [258]. Further, the *SMARCB1* R37H missense mutation does not impact BAF nucleosome remodelling activity in vitro [258, 259]. Of note, the WHD is isolated from the *SMARCB1* C-terminus in the canonical BAF complex but has been predicted to be repositioned closer to the nucleosome binding lobe in the C-terminal domain in the PBAF complex [260, 272]. This is suggestive of a different functional impact for the *SMARCB1* R37H mutation in the different BAF complexes (BAF vs. PBAF).

### Reduced Schwannoma risk in patients with 22q11.2 deletions

Of the clinical syndromes associated with germline deletions at 22q11.2, the best characterized is the proximal chromosome 22q11.2 microdeletion syndrome, leading to DiGeorge syndrome/velocardiofacial syndrome (DGS) [MIM #188400 ] most often caused by deletions of ∼3 Mb spanning LCR22 A–D [reviewed by 195] (Fig. 2). Of note, these 3-Mb deletions include the *LZTR1* gene but not *SMARCB1*. Remarkably, patients with these 3-Mb deletions have a lower risk for developing schwannomas as compared to the general population [398]. This is most likely due to the observation that in patients with germline whole-gene deletions of a tumour suppressor gene, the second tumour-specific mutational event is never loss-of-heterozygosity (LOH) of the wild-type allele of this tumour suppressor gene but rather invariably a subtle intragenic mutation [399]. In patients with 3-Mb deletions and DGS, LOH leading to loss of *LZTR1* and *NF2* as well as flanking regions or larger parts of 22q would appear to be impaired. However, as outlined in Sect. Co-involvement of several tumour suppressor genes in schwannoma development, *NF2* loss is indispensable for schwannoma growth. Hence, the 3-Mb deletion in proximal 22q11.2 causing DGS appears to confer a reduced risk for schwannoma development [398]. Importantly, schwannomas have not so far been reported in patients with distal 22q11.2 deletions and increased risk for MRTs. Most likely, somatic LOH caused by mitotic recombination leading to the loss of large parts of 22q, including the *NF2* gene, is impaired not only in patients with proximal 22q11.2 deletions but also in those with distal 22q11.2 deletions that encompass the *SMARCB1* gene.

## Conclusion

The important role of the BAF complex during cellular and tissue differentiation, in particular nervous system development, provides a link between tumour suppression and neurodevelopment. Chromatin remodelling is essential for the differentiation of the neural crest. Impairment of proper chromatin remodelling may give rise to neural crest-derived tumours or neurodevelopmental disorders. The exit of neural crest cells from pluripotency towards lineage-specific differentiation would appear to be particularly vulnerable to BAF complex dysfunction, including SMARCB1 loss-of-function in a dosage-dependent manner.

The functional impairment of the SMARCB1 protein or other BAF complex subunits during neural differentiation may either lead to aberrant proliferation and tumorigenesis or intellectual disability and developmental delay. Germline *SMARCB1* PVs are associated with RTPS1 and the development of malignant rhabdoid tumours, schwannomatosis or neurodevelopmental disorders such as CSS and ID-CPH. Several factors appear to determine the type of pathology associated with germline *SMARCB1* PVs. First, the timing of *SMARCB1* inactivation, either during early embryonic development or during later stages in specific progenitor cells, is an important determinant. Several model systems have indicated a very early developmental window for the origin of pediatric MRTs caused by a specific vulnerability to biallelic *SMARCB1* inactivation in early neural crest cells. By contrast, schwannomas are likely to result from more differentiated cells such as Schwann cell precursors, which are migrated multipotent progenitors causing schwannoma growth in different body locations during later life. 

Second, the different pathologies caused by *SMARCB1* mutations are strongly influenced by the type of the germline *SMARCB1* pathogenic variant leading to either complete loss of *SMARCB1* function in MRTs or a semi-functional SMARCB1 protein resulting from a pathogenic but hypomorphic *SMARCB1* variant in patients with schwannomatosis. The type and position of the germline PV within *SMARCB1* would also appear to play an important role in the context of *SMARCB1*-associated neurodevelopmental disorders such as CSS. The majority of heterozygous *SMARCB1* PVs causing CSS are single residue alterations located in the C-terminal domain of *SMARCB1*, which serve to impair the interaction of the BAF complex with the nucleosome. However, these PVs do not interfere with the tumour suppressor functions of *SMARCB1*. Thus, the CSS-causing *SMARCB1* PVs may specifically affect central nervous system development but do not cause malignancy in patients with CSS.

A third determinant of the different pathologies caused by *SMARCB1* PVs is represented by additional genomic and epigenetic changes. The mutation type associated with the loss of the second *SMARCB1* allele (intragenic *SMARCB1* PV, large deletion, complete loss of chromosome 22q) may also influence tumorigenesis. Whilst biallelic *NF2* gene inactivation is an absolute requirement for schwannoma growth in addition to biallelic *SMARCB1* mutation, complete *NF2* gene inactivation is dispensable for MRT development. Malignancy in MRTs is driven by massive changes in the epigenome due to *SMARCB1* loss accompanied by changes in the expression of hundreds of genes leading to an undifferentiated tumour phenotype with a very poor prognosis. The continuing analysis of the multifaceted roles of *SMARCB1* in cell cycle regulation, DNA repair, gene activation and repression, neurogenesis and nervous system development promises to identify further determinants of *SMARCB1*-associated pathologies.

## Electronic supplementary material

Below is the link to the electronic supplementary material.

Supplementary Material 1

## Data Availability

No datasets were generated or analysed during the current study.
