# Supplementary material for: SMARCB1-related schwannomatosis and other SMARCB1-associated phenotypes: clinical spectrum and molecular pathogenesis
Source: Fam Cancer. 2025 Aug 12;24(3):64. doi: 10.1007/s10689-025-00486-4 (PMC12343709; doi:10.1007/s10689-025-00486-4)
Supplement: Supplementary file 1 — Supplementary Material 1 [file 10689_2025_486_MOESM1_ESM.docx]

**Supplementary Table 1: Diagnostic criteria for schwannomatosis according to Baser et al. [64]**

| **Definite schwannomatosis** |
| --- |
| 1. Age > 30 years plus two or more non-intradermal schwannomas, at least one with histological confirmation   **OR** |
| 1. One pathologically confirmed schwannoma plus a first-degree relative who meets the above criteria |
| **Possible schwannomatosis** |
| 1. Age < 30 years plus two or more non-intradermal schwannomas, at least one with histological confirmation   **OR** |
| 1. Age >45 years plus two or more non-intradermal schwannomas, at least one with histological confirmation   **OR** |
| 1. Radiographic evidence of a schwannoma and a first degree relative meeting the criteria for definite schwannomatosis |

All individuals must fail to meet the diagnostic criteria for neurofibromatosis type 2 (NF2), in particular must not have vestibular schwannomas as determined by high resolution MRI, must lack a first degree relative with NF2 and must not harbour a germline pathogenic variant in the *NF2* gene.

**Supplementary Table 2: Potential schwannomatosis-predisposing genes**

| **Gene** | **MIM#** | **Chromosomal localization** | **Reference** |
| --- | --- | --- | --- |
| *MYO18B***^a^** | 607295 | 22q12.1 | [43] |
| *NEFH***^a^** | 162230 | 22q12.2 | [43] |
| *SGSM1***^a^** | 611417 | 22q11.23 | [43] |
| *SGSM3***^a^** | 610440 | 22q13.1-13.2 | [43] |
| *SBF1***^a^** | 603560 | 22q13.3 | [43] |
| *COQ6***^b^** | 614647 | 14q24.3 | [70] |
| *DGCR8***^c, d^** | 609030 | 22q11.21 | [73, 74] |
| *CDKN2A***^e^** | 600160 | 9p21.3 | [75] |
| *PTCH2^f^* | 603673 | 1p34.1 | [72] |

a: The five potential SWN-predisposition genes reported by Piotrowski et al. [43] were identified by the analysis of 31 patients with 22q-related SWN. This type of SWN is characterized by the absence of identifiable germline *LZTR1* or *SMARCB1* PVs. In schwannomas, however, somatic 22qLOH with or without an identifiable somatic *NF2* PV is detected in patients with 22q-related SWN [66]. In an attempt to identify additional schwannomatosis predisposition genes on chromosome 22, Piotrowski et al. [43] performed targeted sequencing of specifically chromosome 22q in the 31 patients with 22q-related SWN. The five genes on chromosome 22 identified in this study as harbouring pathogenic variants may in time qualify as additional schwannomatosis predisposition genes, but further verification is currently required.

b: Zhang et al. [70] analysed eight individuals with schwannomatosis from four consecutive generations of the same family. All affected family members were negative for germline *NF2*, *SMARCB1* and *LZTR1* pathogenic variants. Schwannomas of affected family members were not genetically analysed. The *COQ6* PV in exon 6 (c.622G>C; p.Asp208His) was identified by whole-genome/exome sequencing.

c: Rivera et al. [73] investigated a three-generation family with euthyroid multi-nodular goiter and schwannomatosis. The affected patients were negative for germline *NF2*, *SMARCB1* and *LZTR1* pathogenic variants. In the schwannomas from the patients, 22qLOH was detected and hence they can be classified as having a form of 22q-related SWN. By means of whole-genome sequencing, a germline c.1552G>A; p.Glu518Lys variant in exon 7 of *DGCR8* was identified in affected family members.

d: Nogué et al. [74] reported a patient with a mutational pattern of 22q-related SWN, and a pathogenic missense variant in the *DGCR8* gene (c.1552G>A; p.E518K) as identified by whole-exome sequencing. The patient had sporadic schwannomatosis and no thyroid nodules.

e: Perez-Becerril et al. [75] identified a single nucleotide duplication in exon 2 of *CDKN2A* (g.28291dup) in a patient with multiple schwannomas. The duplication disrupted both isoforms of *CDKN2A*, encoding p16INK4a (NM_000077.4; c.158dup; p.Met53fs) and p14ARF (NM_058195.3; c.201dup; p.Asp68Ter), respectively. The patient did not possess germline PVs in *NF2*, *SMARCB1* or *LZTR1*. Nor was 22q LOH detected in schwannoma tissue from this patient.

f: Min et al. [72] identified the frameshift deletion (c.1170_1171del; p.Ser391 fs) in the *PTCH2* gene in two unrelated sporadic patients with multiple schwannomas who were negative for germline *NF2*, *SMARCB1* and *LZTR1* PVs by means of whole-exome sequencing.

**Supplementary Table 3: Germline pathogenic variants (PVs) and a variant of unknown significance (VUS) located in deep intronic regions or 3’UTR sequences within the *SMARCB1* gene identified in patients with *SMARCB1*-related SWN or patients with AT/RT**

| **Variant in *SMARCB1*** | **cDNA variant (RNA variant)**  **[protein variant]** | **Comment** | **References** |
| --- | --- | --- | --- |
| Deep intronic PV | c.500+887G>A  c.500+883T>G | Variant confirmed by RNA analysis to result in mis-splicing of intron 4 and the insertion of a premature stop codon | [43] |
| Deep intronic, probable PV | c.629-2818C>T | No effect on splicing as determined by cDNA-based Sanger sequencing analysis. | [43] |
| Deep intronic PV | c.795+1498C>T (r.795+1403_795+1496)  [p.(Lys265Lysfs*46)] | Variant leads to an out‐of‐frame insertion of 94 nucleotides resulting in a premature stop codon. It was present in all 5 affected family members but was absent from an unaffected family member; clinical variability was evident between affected family members. | [81, 370, 372] |
| Deep intronic PV | c.93+559A>G  (r.93_94ins93+483_93+554) | Sequencing of intron 1 revealed a variant c.93+559A>G in the genomic DNA, producing a new donor splice site and exonisation of a part of intron 1. Sequencing of cDNA indicated an insertion of 72 nucleotides from intron 1 between exons 1 and 2 which resulted in the formation of a premature termination codon. This variant was present in two unrelated individuals (4-months and 8-months old respectively) with atypical teratoid rhabdoid tumours; variant was absent from the unaffected parents, suggestive of the *de novo* occurrence of the variant or the presence of gonadal mosaicism in one parent. | [371] |
| VUS in the 3’ UTR | c.*70C>T | Variant detected in a 45-year-old woman with painful spinal schwannomas located in her abdomen and neck; a positive family history of schwannomas in a child and two grandchildren; the child tested positive for the *SMARCB1* c.*70C>T variant. Expression analysis of this variant indicated that it does not affect the *SMARCB1* transcript level. Therefore, the variant was classified as a variant of unknown significance (VUS). The variant was identified in tumours excised from two additional unrelated schwannomatosis patients as a somatic alteration not present in the germline. | [43] |
| PV in the 3’UTR | c.*17C>T | Variant negatively affected the *SMARCB1* transcript level; observed in a male SWN patient with schwannomas detected at the age of 8. | [43] |
| PV in the 3’UTR | c.*82C>T | Variant adversely affects *SMARCB1* transcript stability, resulting in reduced levels of the transcript with the PV, as compared to the transcript with the wild‐type 3′‐UTR; it is the most common recurrent PV observed in patients with *SMARCB1*-related SWN. | [34, 38, 40, 42, 43, 81, 362, 363] |
